# Phytochemicals of Avocado Residues as Potential Acetylcholinesterase Inhibitors, Antioxidants, and Neuroprotective Agents

**DOI:** 10.3390/molecules27061892

**Published:** 2022-03-15

**Authors:** Geisa Gabriela da Silva, Lúcia Pinheiro Santos Pimenta, Júlio Onésio Ferreira Melo, Henrique de Oliveira Prata Mendonça, Rodinei Augusti, Jacqueline Aparecida Takahashi

**Affiliations:** 1 Department of Food Science, Faculty of Pharmacy, Universidade Federal de Minas Gerais, Av. Antônio Carlos, 6627, Belo Horizonte 31270-901, Brazil; geisambio@gmail.com; 2 Chemistry Department, Universidade Federal de Minas Gerais, Av. Antônio Carlos, 6627, Belo Horizonte 31270-901, Brazil; lpimenta@qui.ufmg.br (L.P.S.P.); augusti.rodinei@gmail.com (R.A.); 3 Exact and Biological Sciences Department, Campus Sete Lagoas, Universidade Federal de São João del-Rei, Sete Lagoas 36307-352, Brazil; onesiomelo@gmail.com (J.O.F.M.); hp.quimico@hotmail.com (H.d.O.P.M.)

**Keywords:** avocado biomass, Alzheimer’s disease, neuroprotective effect, avocado seed, avocado peel, paper spray mass spectrometry

## Abstract

Avocado (*Persea americana*) is a widely consumed fruit and a rich source of nutrients and phytochemicals. Its industrial processing generates peels and seeds which represent 30% of the fruit. Environmental issues related to these wastes are rapidly increasing and likely to double, according to expected avocado production. Therefore, this work aimed to evaluate the potential of hexane and ethanolic peel (PEL-H, PEL-ET) and seed (SED-H, SED-ET) extracts from avocado as sources of neuroprotective compounds. Minerals, total phenol (TPC), total flavonoid (TF), and lipid contents were determined by absorption spectroscopy and gas chromatography. In addition, phytochemicals were putatively identified by paper spray mass spectrometry (PSMS). The extracts were good sources of Ca, Mg, Fe, Zn, ω-6 linoleic acid, and flavonoids. Moreover, fifty-five metabolites were detected in the extracts, consisting mainly of phenolic acids, flavonoids, and alkaloids. The in vitro antioxidant capacity (FRAP and DPPH), acetylcholinesterase inhibition, and in vivo neuroprotective capacity were evaluated. PEL-ET was the best acetylcholinesterase inhibitor, with no significant difference (*p* > 0.05) compared to the control eserine, and it showed neither preventive nor regenerative effect in the neuroprotection assay. SED-ET demonstrated a significant protective effect compared to the control, suggesting neuroprotection against rotenone-induced neurological damage.

## 1. Introduction

Originated from Central America, the avocado is the fruit of the *Persea americana* Mill species from the Lauraceae family. It is rich in minerals, vitamins, proteins, fibers, and unsaturated lipids, the latter known to prevent cardiovascular diseases [1,2]. Moreover, avocado seeds display significant antioxidant, anti-inflammatory, and anti-cancer effects which have been associated with elevated percentages of hydrocarbons, sterols, and unsaturated fatty acids. Pulp has the same effects, although at a lower intensity [3]. Finally, the peel is rich in phenolic compounds and minerals, and has significant antioxidant activity, comparable to the seeds and far superior to the pulp [4,5]. 

In 2020, Mexico was the largest avocado producer, accounting for 2393 thousand tons per year, followed by the Dominican Republic, Peru, Colombia, Indonesia, and Brazil [6]. Since the inedible parts of avocado, mainly constituting peels and seeds, are discarded by the industry, restaurants, or at home, with the growth of avocado production, the amount of residues is also increasing, causing environmental problems [7]. These parts can reach up to 30% of the fruit. Furthermore, it is predicted that avocado production will double shortly [8]. In this sense, changes in the management of this fruit are necessary to accompany the dynamics of economic demands, with minimal environmental impacts [8,9,10]. Because avocado solid waste retains useful bioactive natural compounds, this residue has been targeted by several interesting studies to recover phytochemicals of industrial interest [11,12,13,14]. 

The antioxidant activity of avocado inedible parts can be explored in the development of neuroprotective agents against oxidative-related diseases like Alzheimer’s [4,5,11,14]. Alzheimer’s disease is one of the most concerning neurodegenerative disorders, affecting over 55 million people, mainly the elderly [15]. This disease can disrupt the synapses between neurons by the action of acetylcholinesterase (AChE), an enzyme that prevents acetylcholine from establishing communication between neurons. There is still no cure for this pathology, but palliative drugs have been very useful in decreasing symptoms and improve the quality of life of patients with Alzheimer’s disease. However, these drugs are usually expensive. The monthly cost of galantamine (30 pills) almost reaches USD 200, while drugs for the treatment of other diseases such as diabetes and hypertension cost less than USD 20 [16]. Therefore, the development of new drugs to treat neurodegenerative diseases from avocado biowaste could widen access to treatment while generating economic gains for the industry. 

In this context, this work aimed to validate the nutritional and functional value of avocado residues by carrying out chemical and biological assays on seeds and peels of the Fortuna variety. The mineral, fatty acid, phenolic, and flavonoid contents were determined using spectroscopic and chromatographic methodologies. The phytochemical profile was determined by Paper Spray Mass Spectrometry (PSMS). Biological assays targeted antioxidant potential, AChE inhibition, and neuroprotective efficacy.

## 2. Results and Discussion

### 2.1. Residues Weight and Extracts Yields

Avocado fruits were separated into parts and weighted, resulting in 69.9% pulp, 10.2% peel, and 19.9% seed. According to the FAO [17], the world production of avocado is estimated to reach 9.2 tons by 2028. In this scenario, the volume of residues can reach approximately two tons. The peels were extracted by maceration with hexane and ethanol, providing two extracts, PEL-H (1.41 g/100 g of sample) and PEL-ET (16.94 g/100 g of sample). The same procedure was repeated with the seeds to furnish hexane (SED-H, 0.94 g/100 g of sample) and ethanol (SED-ET, 28.58 g/100 g of sample) extracts. The high yields of ethanol extracts reflect greater extraction power than that of hexane. The substitution of organic solvents with an environmentally favorable solvent, such as ethanol, makes the recycling of avocado waste important in terms of green chemistry and sustainability.

### 2.2. Mineral Contents in Avocado Residues

The mineral contents were quantified by atomic absorption spectroscopy, and the results are presented in Table 1.

According to the results, both avocado peels and seeds are good sources of minerals, especially calcium and magnesium, along with minor amounts of copper, iron, manganese, and zinc. Calcium, together with iron and zinc, are among the most difficult nutrients to obtain using local foods, according to research targeting women and young children from Southeast Asia [18]. In the U.S., mineral supplementation is advised during pregnancy [19]. Women are likely to be more affected by mineral deficiency, especially during childhood, although in many countries like Brazil, the prevalence of mineral deficiency is underreported [20]. Additionally, each ton of an agro-industrial residue containing avocado peels and seeds would correspond to the FAO/WHO’s daily recommended intake of calcium (1024 mg) for over two thousand individuals for one month, which is very considerable given calcium deficiency in some groups. The mineral content of agro-industrial residues is variable. Peels and seeds of avocado have lower mineral content than cocoa honey, a cocoa byproduct [21], but higher than pea peels [22]. Minerals and other nutrient and bioactive compounds present in agro-industrial residues can be processed in different ways, such as snack crackers and dry soup [22].

### 2.3. Fatty Acid Composition

After chemical derivatization to fatty acid methyl esters (FAMEs), the extracts were evaluated by gas chromatography, allowing identification of the fatty acids presented in Table 2. 

Linoleic acid, a ω-6 fatty acid, was the component predominant in the seed extracts (27.4 and 34.8%) and palmitic acid, the major component (42.5–47.9%), in the seed extracts (22.2–23%). Oleic acid was distributed in all samples, but its content was lower in the peel ethanolic extract. Stearic acid was detected in all samples, but mainly in the ethanol extracts (14.7% in the seeds/22.2% in the peels). The composition of the different extracts revealed that seeds had a higher content of unsaturated fatty acids, such as oleic and linoleic, and small amounts of saturated fatty acids, such as palmitic and stearic.

The human body cannot synthesize linoleic acid, an essential fatty acid; therefore, it must be acquired through feeding. Linoleic acid plays a fundamental role in fetal neurological function and infant growth because it is the precursor of arachidonic acid. The uptake of this fatty acid decreases the risk of developing heart disease and it is beneficial for inflammatory processes [23]. Furthermore, palmitic and stearic acids are undesirable in food because saturated fatty acids usually contribute to increased blood cholesterol. However, stearic acid is considered an exception, because a rapid in vivo enzymatic dehydrogenation reaction catalyzed by stearoyl-CoA Δ9-desaturase converts stearic acid to oleic acid (18:1 Δ9) in the body [24]. The fatty acid composition found in avocado residue extracts suggests that they could be better used in combination with other oils, to balance the final composition. For instance, a sample of olive oil studied by Orsavova et al. [25] presented 16.4% of linoleic acid and 16.5% of palmitic acid (~1:1 proportion). A 1:1 mixture of this olive oil and the oil present in the SED-H would increase the proportional quantity of linoleic acid to 26% and the amount of palmitic acid to 19.75%. In the end, such mixed oil, composed of 50% oil recovered from waste biomass, would benefit from an improved content of linoleic acid. 

The health claims for avocado fruits are closely related to the high content of fatty acids that are essential for human health [26]. Therefore, the essential fatty acids detected in the residues already consist of valuable metabolites that could be used as food additives or might be extracted, adding value to avocado waste biomass, which is a promising and inexpensive alternative source of linoleic acid. 

### 2.4. Determination of the Total Phenolic and Flavonoid Contents 

Avocado is a rich source of polyphenols, including hydroxybenzoic acid, caffeoylquinic acid derivatives, and cinnamic acids [5,27]. Flavonoids, such as quercetin, quercetin glycosides, naringenin, catechin, and epigallocatechin, were described in avocado seeds of the “Hass” variety [5]. In the current study, the total phenolic content (TPC) was estimated using the Folin–Ciocalteu method for each extract and expressed in mg of gallic acid equivalents (GAE)/g of extract. The total flavonoid content (TFC) was determined using the aluminum chloride colorimetric method and expressed as miligram of quercetin per gram of extract. Table 3 presents the results of the polyphenol and flavonoid contents according to the previous methodologies mentioned. 

The TPC values was found in the range of 26.33–35.40 mg of gallic acid/g of extract, and the TFC values ranged from 640.72 to 1199.04 mg of quercetin/g of extract. The peel ethanolic extract (PEL-ET) presented the highest content of total phenolic compounds (35.40 ± 0.599 mg of gallic acid/g of ethanol extract), which was significantly different from all other extracts. Similarly, the TPC reported for peels from another variety of avocados using the same methodology (Folin-Ciocalteau) was 47.9 ± 2.7 mg of gallic acid/g of ethanol extract [28]. Conversely, peel ethanolic extracts from different varieties of *P. Americana*, including the Fortuna variety, presented lower phenolic compounds contents [29].

The peel extracts presented significantly higher flavonoid content than the extracts of the corresponding seeds. Higher percentages of flavonoid compounds were expected in the ethanol extracts, given the polar nature of the most representative flavonoids. However, our results showed that the higher percentages were present in the hexane extracts. This discrepancy can be partially explained by the presence of vitamin E in avocado residues [2]. Vitamin E is a phenolic non-polar compound, known to be present in substantial levels in avocado. Probably, its aliphatic long side chain may have contributed to the results. In addition, the percentages were determined by a colorimetric method that, although being a widely used methodology, is not a specific analysis. Flavonoid compounds have also been identified in the peels and seeds of the Hass and Fuerte varieties using HPLC-DAD [30].

### 2.5. Total Antioxidant Capacity and Antioxidant Activity Determined by DPPH Radical Scavenging and Ferric Reducing Power

The results regarding the antioxidant functional properties are shown in Table 4. In the total antioxidant activity assay (expressed as mmol of ascorbic acid/g extract) (R² = 0.9339), the values ranged from 630.23 to 770.01 and all extracts from seeds (SED-H and SED-ET) and peel hexane extracts (PEL-H) showed no significant differences. 

Peel and seed hexane extracts presented similar behavior in promoting the reduction of ferricyanide ion to ferrocyanide, forming Prussia’s blue. The reducing power of both seed and peel ethanolic extracts was lower. The extract capacities of scavenging DPPH radicals were, in general, low. Only the PEL-ET showed moderate activity presenting over 50% of inhibition, with an IC_50_ 92.557 μg/mL, being, therefore, the most promising extract in this assay. The high antioxidant activity may be explained by the high content of phenolic compounds, which was observed for peel and seed ethanolic extracts.

### 2.6. Acetylcholinesterase Inhibition

**Figure 1:**
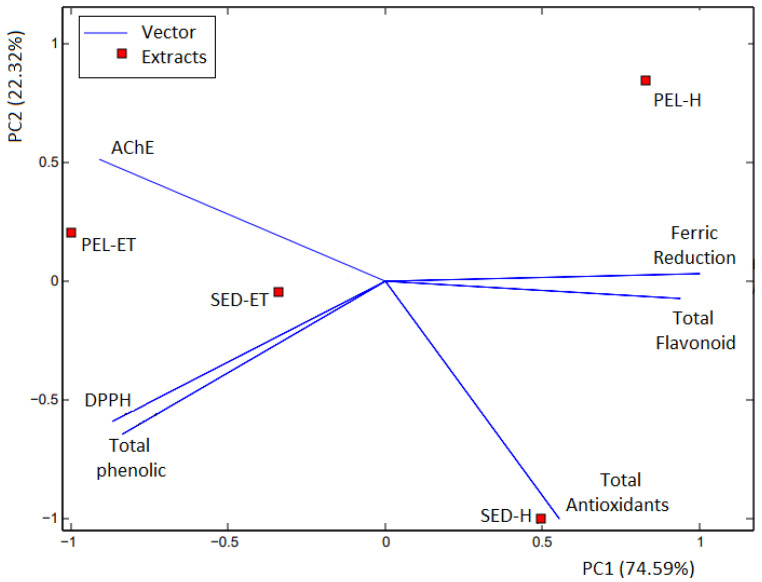
Principal component analysis biplot, PC1 versus PC2, correlating antioxidant responses, total phenolic content, total flavonoid content, and acetylcholinesterase (AChE) inhibitory activity. DPPH: 2,2-Diphenyl-1-picrylhydrazyl; SED-H: seed hexane extract; SED-ET: seed ethanolic extract; PEL-H: peel hexane extract, and PEL-ET: peel ethanolic extract.

The hexane (SED-H and PEL-H) and ethanolic (SED-ET and PEL-ET) extracts were submitted to acetylcholinesterase inhibition assay. All extracts inhibited acetylcholinesterase activity up to 65%, with the ethanolic extracts more active than the hexane ones (Table 4). This range of extract activities is promising when compared with the activity presented by the control, a pure compound. The extract is a complex mixture and the active constituents are usually minor constituents in the extracts. In this way, the inhibitory capacity of the peel ethanolic extract from avocado (85.6 ± 11.1%) over acetylcholinesterase is outstanding, presenting no significant difference (*p* > 0.05) compared to the activity of the pure standard, eserine (91.5 ± 1.6%). Comparing the whole picture, the extract PEL-ET demonstrated a direct relationship between the amount of total phenolic compounds and AChE inhibition, corroborating the influence of antioxidant phenolic compounds in the acetylcholinesterase activity. By contrast, an inverse relationship was perceived for the hexane and ethanol extracts of the seeds. The principal component analysis (PCA) score biplot allowed a general qualitative visualization of the results obtained in the biological screening (Figure 1).

The PCA biplot shows the close relationship of the ethanolic extracts with the presence of phenolic compounds, DPPH assay, and acetylcholinesterase inhibition. The peel ethanolic extract (PEL-ET) demonstrated the closest association with the acetylcholinesterase enzyme inhibition assay. Concerning the hexane extracts, the seed extract (SED-H) was mainly related to the total antioxidant activity, while the peel extract (PEL-H) is associated with both ferric reduction power and total flavonoid contents. The hexane extracts are more distant from the AChE assay and present lower activity for acetylcholinesterase inhibition, as observed in the study. Therefore, only the ethanolic extracts were submitted to the in vivo D. melanogaster assay to evaluate the neuroprotective response. 

### 2.7. Activity of P. americana Extracts in Neuroprotection Using D. melanogaster Model 

*D. melanogaster* flies have been used as an in vivo model for the evaluation of neurological damage related to neurodegenerative conditions. In this model, neurological damage can be induced in the flies by in vitro feeding with chemicals such as rotenone, preventing these insects from flying. The *D. melanogaster* model was used by Jiménez et al. [31] to demonstrate the neuroprotective effect of methanol extract prepared from *Solanum ovalifolium*, and by Siima et al. [32] to show the effect of flavonoids and polyketides in rescuing locomotor capacity in a Parkinson’s disease-related study [33]. Test compounds can be administered before or after flies’ exposure to rotenone to evaluate protective and regenerative effects, respectively. In the first alternative, the test compound avoids some of the damages that rotenone would cause (preventive action). In the second, damages due to rotenone exposure are established in the flies before test compound administration, and the capacity of the target compound to revert the neurodegenerative effects is evaluated (regenerative action). 

**Figure 2:**
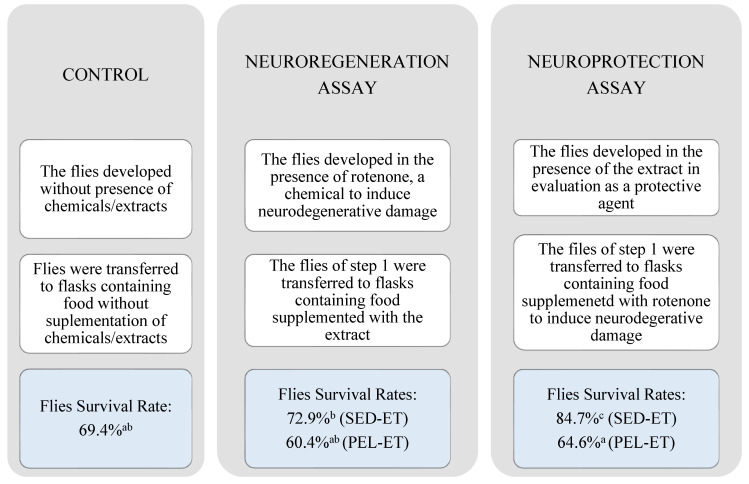
Outline and results of negative geotaxis assay of peels and seeds ethanol extracts in comparison with the control. Different letters indicate statistical difference by Dunn’s test (*p* < 0.05). SED-H: seed hexane extract; SED-ET: seed ethanolic extract; PEL-H: peel hexane extract, and PEL-ET: peel ethanolic extract.

In the present work, test compounds were administered before and after exposure to rotenone, to evaluate both effects (Figure 2). The ethanol extracts (PEL-ET and SED-ET) were evaluated by this model and the results were submitted to the Shapiro–Wilk statistical normality test. The *p*-value found (>0.05) indicated that the distribution was not suitable. Therefore, the Kruskal–Wallis nonparametric test was conducted, identifying a significant difference; subsequently, the Dunn’s test was used, which identified a significant difference *p* < 0.05 between the samples. Overall, a high mortality rate was observed during the trial in which flies were first exposed to rotenone (Figure 2). In this condition, rotenone caused 60.4–72.9% of flies to die, showing high acute toxicity. Under this condition, peel extract (PEL-ET) did not statistically differ (*p* > 0.05) from the control, in which flies were not exposed either to the extracts or to rotenone. Therefore PEL-ET showed neither preventive nor regenerative effect in this model. 

SED-ET showed a more significant result (Figure 2). Comparing the seed and peel results in this assay, only SED-ET demonstrated a protective effect in this treatment. In addition, the performance of SED-ET was significant compared to the control, presenting a higher number of flies capable of flying above the limit established, which suggests that the seed extract provided a neuroprotective effect against rotenone-induced neurological damage (Figure 2). This flies model has been leading to the identification of new therapeutic targets for research in Parkinson’s [34], Alzheimer’s [35], and other neurodegenerative diseases. The regenerative activity has been the main target of drugs to individuals in the advanced neurodegeneration stage. However, protection against degeneration can be helpful to patients in the early stages of neurodegenerative diseases.

### 2.8. Identification of Metabolites by Paper Spray Mass Spectrometry

**Figure 3:**
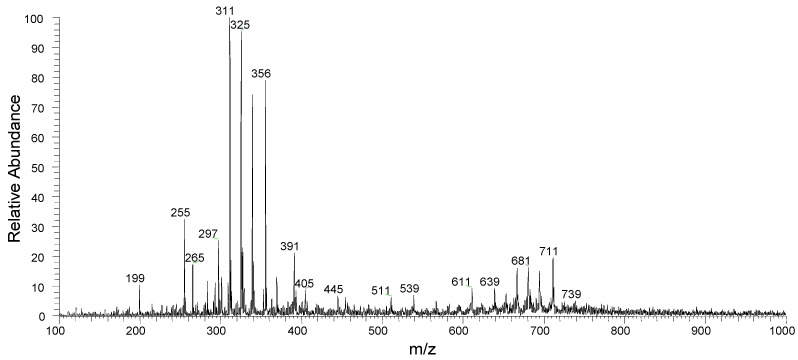
PS (−) MS full scan of ethanol extracts from seed of avocado.

Paper spray mass spectrometry (PSMS) was applied to identify the natural products present in the ethanol extracts. This quick and efficient technique for chemical profile determination is commonly used in the clinic and in forensic research to identify specific ions through direct sample analysis [36,37,38,39]. This study showed no difference in the chemical profile of peel and seed samples. PS (−) MS spectrum of SED-ET is presented in Figure 3. Fifty-five compounds were tentatively identified in the ethanol extracts by PSMS analyzing their MS and MS2 spectra, together with information previously reported in the literature. In the negative mode, 41 metabolites were identified, mainly flavonoids and organic acids, most of them with biological activities already reported [12]. 

The presence of compounds such as caffeic acid (*m*/*z* 179 [M − H]^−^), catechin (*m*/*z* 289 [M − H]^−^), rutin (*m*/*z* 609 [M − H]^−^), procyanidin isomers, and flavonoids validated the antioxidant activity of the seed extract and were in accordance with previous reports [39,40]. Corroborating the experiments, some of the metabolites detected in the current work were already reported in the seeds and peels of avocado (vanillin, caffeic acid, ferulic acid, synaptic acid, kaempferol, catechin, quercetin-3-glucoside, quercetin-O-arabinosyl-glucoside, rutin, dimers A and B, and trimer A of procyanidin) [12]. Based on the antioxidant effect, Segovia et al. [41] described the role of avocado seed extract as an additive to protect oil mixtures from oxidation. The presence of quinic acid derivatives in the extract also contributed to validating the antioxidant activity and, therefore, highlighted the beneficial properties of this residue as food ingredients for industrial use [42]. Table 5 presents the compounds tentatively identified in the extracts in negative mode.

**Figure 4:**
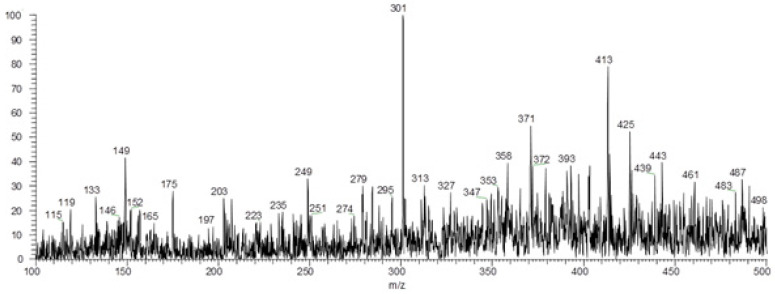
PS (+) MS full scan of ethanol extract from seed of avocado.

**Figure 5:**
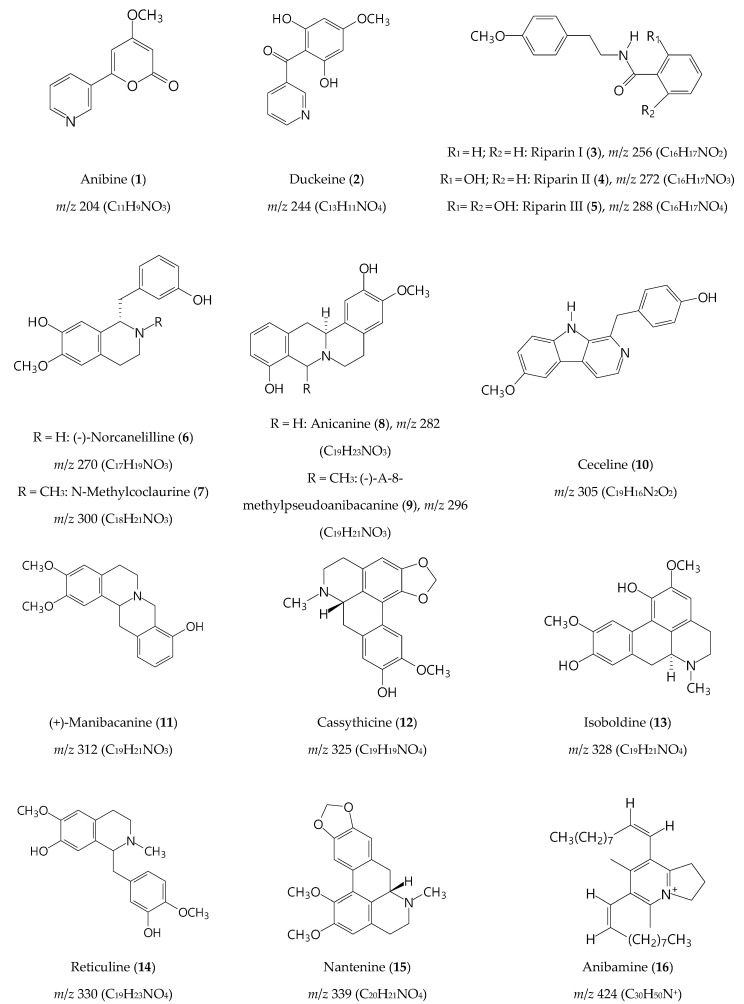
Chemical structures, *m*/*z* and chemical formulas of alkaloids putatively identified by PSMS in seed and peel of avocado.

In the positive mode, fifteen alkaloids were identified: anibine (**1**), duckeine (**2**), riparin I, II and III (**3**–**5**), norcanelilline (**6**), N-methylcoclaurine (**7**), anicanine (**8**), (-)-α-8-methylpseudoanibacanine (**9**), ceceline (**10**), (+)-manibacanine (**11**), cassythicine (**12**), isoboldine (**13**), reticuline (**14**), nantenine (**15**), and anibamine (**16**) (Figure 4 and Figure 5, Table 6). PS(+)MS full scan of ethanol extract from peel of avocado can be found in Supplementary Materials, Figure S1. It is reported that, as the avocado fruit develops, the carbohydrate amount decreases and the fatty acids and secondary metabolites content increases [46,47]. In ripe avocado fruits, the alkaloids constitute a remarkable percentage of the natural compounds present in the pulp [46,47]. From the alkaloids identified, anibine (**1**), duckeine (**2**), and anicanine (**8**) were not detected in PEL-ET. All other alkaloids were detected in both extracts.

Alkaloids are used as active substances in prescriptions for the treatment of neurodegenerative diseases, e.g., galantamine. Therefore, the alkaloids detected in the extracts may contribute to the acetylcholinesterase inhibition and neuroprotective effect. More specifically, the alkaloids riparin (**3**–**5**) show antioxidant, antinociceptive, anti-inflammatory, and neuroprotective effects [49,50,51]. The aporphine alkaloid nantenine (**14**) has a potential role in acetylcholinesterase inhibition, since nantenine is present in *Unopsis stipitate*, a plant traditionally used for cognitive disorders [52]. Furthermore, nantenine possesses pharmaceutical interest as anticonvulsant, due to its direct inhibitory effect on calcium influx [53]. 

## 3. Materials and Methods

### 3.1. Materials and Reagents 

Ripe avocado (cultivar *Persea americana* var. Fortuna) fruits were purchased in Ibirité city (MG, Brazil) in the winter of 2018. P.A. grade hexane (Nox Lab Solutions, Mauá, SP, Brazil) and hydrated ethanol 96% (Emfal, Betim, MG, Brazil) were used. Ascorbic acid (Neon, Suzano, SP, Brazil), gallic acid (Neon, Suzano, SP, Brazil), quercetin (Sigma-Aldrich, St. Louis, MO, USA), and eserine (Sigma-Aldrich, St. Louis, MO, USA) were used as positive controls in the biological assays. Acetylcholinesterase iodide, acid 5′,5′-dithio-bis-(2-nitrobenzoate), bovine serum albumin, and AChE (Sigma-Aldrich, St. Louis, MO, USA) were used in the AChE inhibition assay. For cultivation of *Drosophila melanogaster* flies, bacteriological agar (Vetec, Duque de Caxias, SP, Brazil), powdered dry yeast (Fleischmann, Sorocaba, SP, Brazil), and nystatin (oral suspension from Germed, Campinas, SP, Brazil) were used. Rotenone (Sigma-Aldrich, At. Louis, MO, USA) was employed in the negative geotaxis assay to induce toxicity. The experiments were carried out in quintuplicate unless otherwise specified. 

### 3.2. Biomass Processing and Extract Preparation 

The peels (53.233 g) and seeds (103.431 g) of avocado fruits were separately removed from the pulp, sanitized under running water, and dried separately with paper towel sheets. The peels were sliced, the seeds were grated, and 3 g of each was utilized for atomic absorption spectroscopy analysis. The remaining biomasses (sliced peels and grated seeds) were separately soaked in hexane (600 mL). After 24 h, the hexane fraction was separated by filtration and another aliquot of hexane (600 mL) was added to the vegetal material. The procedure was repeated three times. The hexane fractions were combined; the solvent was removed in a rotary evaporator (60 °C), obtaining the respective hexane extracts of peels (PEL-H) and seeds (SED-H). After hexane extraction, the defatted plant material was further extracted with 96% ethanol, following the same procedure previously described, to furnish the peel (PEL-ET) and seed (SED -ET) ethanolic extracts (Figure 5) [54].

### 3.3. Mineral Element Analysis by Atomic Absorption 

The separated aliquots of fresh peels and seeds (3 g each) were placed in a porcelain crucible and heated until complete carbonization. Subsequently, the crucibles with the carbonized plant material were placed in a muffle at 550 °C, obtaining the organic matter-free ashes. Nitric acid (2 mL) was added to an aliquot of the ashes (0.05 mg) and the volume was completed with distilled water to 5 mL. The resulting suspension was analyzed using Atomic Absorption Spectrometer AA 240 FS (Varian, Australia) to quantify the minerals Ca, Cu, Fe, Mg, Mn, and Zn [4].

### 3.4. Fatty Acid Gas Chromatography Analysis 

Aliquots (10 mg) of the extracts were derivatized to fatty acid methyl esters (FAMEs) [55], solubilized in methanol, and injected into an HP5890 Gas Chromatograph. A Supelcowax-10 analytical column (30 m × 0.2 mm × 0.2 μm) (Supelco) was used for analysis under the following chromatographic conditions (split 1/50): 150 °C, 0 min, 10 °C /min up to 240 °C and detector at 250 °C. Hydrogen was used as carrier gas (4 mL/min) and the injection volume was 1 μL. A standard of fatty acid methyl esters (FAME C14-C22) were injected, and their retention times were used to comparatively identify the fatty acids present in the avocado residues. 

### 3.5. Total Content of Phenol and Flavonoids Assay 

The determination of total phenolic content was performed spectrophotometrically with a Folin–Ciocalteau reagent method previously described [56]. Briefly, the ethanolic extract solutions (0.5 mL) were mixed with Folin–Ciocalteau reagent (2.5 mL). The solutions were incubated for 3 min at room temperature, and 0.3 mL of saturated sodium carbonate solution was added to each one. After a further incubation period (20 min at 25 °C), absorbance was read at 760 nm using a Biospectro SP-22 Spectrophotometer. The same procedure was carried out for gallic acid (standard), which was used for constructing the calibration curve (10–100 µg/mL; R² = 0.9291). In the equation of the calibration curve established using gallic acid, “y” was replaced by the absorbance and divided by the mass of the extract/standard. The results were expressed in mg gallic acid/g extract. 

The total flavonoid contents were determined by aluminum chloride spectrophotometric method as described by Yang et al. [27]. Briefly, 2.5 mL of the ethanolic extract or standard solutions were mixed with 0.3 mL sodium nitrate solution (5% *w*/*v*) with subsequent addition of aluminum chloride solution (10% *w*/*v*) (3.0 mL). The mixture was vortexed, and 2.0 mL of sodium hydroxide solution (1 M) was added and well vortexed. After 10 min, the absorbances were determined at 510 nm using a Biospectro SP-22 Spectrophotometer. Quercetin standard solutions (350–600 μg/mL) were used for constructing the calibration curve (R² = 0.9887). The result was expressed as mg of quercetin equivalent per g of extract. 

### 3.6. Antioxidant Activity 

Ethanol and hexane extracts (SED-ET, PEL-ET, SED-H, and PEL-H) and the standards (ascorbic acid and gallic acid) were dissolved in ethanol (500 μg/mL), except quercetin (1000 μg/mL). All assays were performed in quintuplicate. Microsoft Excel 2013 software was used for data analysis. 

#### 3.6.1. Total Antioxidant Capacity 

A reagent solution (100 mL) was prepared mixing sulfuric acid (3.22 mL), bibasic phosphate (0.39 g), and ammonium molybdate (0.49 g). An aliquot (0.3 mL) of ethanolic extract solutions (500 µg/mL) was mixed with 3.0 mL of the reagent solution. The resulting test samples were incubated in an oven at 95 °C for 90 min. After the samples had cooled to room temperature, the absorbances were read at 695 nm on a Biospectro SP 22 Spectrophotometer. The same procedure was carried out with ascorbic acid (positive control) and a negative control was carried out with the solvent. The results were expressed in mmol of ascorbic acid per gram of extract (adapted from [57]). 

#### 3.6.2. Ferric Reducing Power Assay 

An aliquot (1.0 mL) of the ethanolic extract solutions was mixed with 2.5 mL of phosphate buffer (pH 6.6, 0.2 M) and 2.5 mL of potassium ferricyanide aqueous solution (1% *w*/*v*). The mixture was homogenized and incubated in a laboratory water bath at 50 °C for 20 min. Subsequently, 2.5 mL of trichloroacetic acid solution (10% *w*/*v*) was added and the mixture was centrifuged (300 rpm, 10 min). An aliquot (2.5 mL) of the superior layer was mixed with 2.5 mL distilled water, and 0.5 mL ferric chloride solution (0.1% *w*/*v*) was added. The same procedure was conducted with the positive control. The absorbances were read at 700 nm on a Biospectro SP-22 Spectrofotometer. The results were converted into a percentage of ferric reducing power in relation to the positive control ascorbic acid [57]. 

#### 3.6.3. Free Radical Capture Assay (DPPH)

Solutions of extracts (100 μL) were added to 96-well plates in quadruplicate, followed by the addition of 2,2-Diphenyl-1-picrylhydrazyl (DPPH) solution (175 μL) prepared in ethanol (0.1 mM). The materials were incubated for 30 min in the dark, after which reading was performed at 490 nm in a microplate reader. Solutions of known concentration of ascorbic acid, the positive control, were used to construct a graph of percent inhibition versus concentration (μg/mL) [58].

### 3.7. Acetylcholinesterase Inhibition Assay

The extracts were solubilized in dimethylsulfoxide (DMSO) (10 mg/mL) and an aliquot of each one (25 μL) was mixed with aqueous solutions of acetylcholine iodide (15 mM) (25 μL) and 125 μL of a solution containing 5,5′-dithio-bis (2-nitrobenzoic acid) (3 mM), NaCl solution (0.1 M) and MgCl_2_·6H_2_O (20 mM) prepared in Tris/HCl buffer (50 mM, pH 8.0, 50 μL) in wells of 96-well plates. The first set of wavelength readings was then performed at 405 nm every 1 min (8 readings). Subsequently, a solution of acetylcholinesterase was prepared (0.22 U/mL) in buffer and 0.1% (*w*/*v*) of bovine serum albumin and added to the wells (25 μL each). Eserine (10 mg/mL) was used as positive control. Next, new readings were performed at the same wavelength every 1 min (10 readings) [20]. The following equation was used to calculate the percentage of inhibition (%*I*), where *Ab*: Negative control (DMSO) and *Aa*: Sample absorbance.

$$\%\;I=\frac{\left(Ab-Aa\right)\times 100}{Ab}$$

### 3.8. In Vivo Neuroprotection Activity Using Drosophila melanogaster Model 

A sterile mixture containing bananas (400 g), bacteriological agar (4 g), commercial yeast (3.5 g), nystatin (2.5 mL), and propionic acid (0.6 mL) in distilled water (350 mL) was prepared and used to grow *D. melanogaster* flies for the whole experiment. The cultivations were performed in glass vessels in triplicate. Twelve 6–8-day-old male flies were grown in vessels containing (A) rotenone (neurotoxic inductor, 10 mg/mL in ethanol; 0.4 mL) or (B) extracts (5 mg/mL in water; 0.6 mL). Rotenone and extracts were mixed with the nutritive substrate. A control flask (C) was utilized. Neither rotenone nor extract were added to the control flasks. After 7 days, the flies that were in vessels A and B were anesthetized with ether and interchanged. After 15 days the flies were transferred to falcon tubes that were horizontally placed on a support. A photographic camera was positioned approximately 30 cm away. Then, the support was subjected to three hits against the bench surface, and ten photographs of the tubes containing the flies were recorded. Picture number 6 was used to account for the number of flies that were able to fly above half the tube height after hitting [31]. 

### 3.9. Paper Spray Mass Spectrometry

Pulps were analyzed using a Thermo Fisher LCQ FLEET ion-trap mass spectrometer (Thermo Scientific, San Jose, CA, USA) equipped with an ambient paper spray ionization source. Analyses were performed in triplicate in positive and negative ionization modes. For PSMS analyses, a 2 µL sample volume and 40 µL of methanol were added to a triangular chromatographic paper (equilateral, 1.5 cm lengthwise) positioned 10 mm away from the mass spectrometer inlet [37,38,39]. A high voltage was then applied to the paper held by a copper clamp and attached to a three-dimensional moving platform for data acquisition. Instrumental conditions of operation were: PSMS source voltage equal to +4 kV (positive mode) and −3 kV (negative mode); capillary voltage of 40 V; transfer tube temperature of 275 °C; tube lens voltage of 120 V; mass scanning range of 100 to 1000 *m*/*z* in positive and negative modes. Ions were fragmented using collision energies from 15 to 45 eV. Some representative MS can be found in Figures S2–S48. Tentative identification of the compounds was carried out using a comparison of the *m*/*z* ratios of the data obtained from literature associated with instrumental readings and subsequent fragmentation employing sequential mass spectrometry.

### 3.10. Statistical Analysis

GraphPad Prism 5.0 software was used for statistical analysis, applying variance analysis (ANOVA) [20]. To evaluate the differences between the means, the Tukey test with significance level of 5% was used. Kruskal–Wallis (nonparametric) and Dunn’s post-test, with significance of 5%, were utilized to evaluate data from neuroprotection assay. SensoMaker 1.61 software was used for main components analyses.

## 4. Conclusions

The avocado residues produced during industrial processing retain nutrients and bioactive compounds independent of the extraction solvent. Concerning the minerals, avocado peels and seeds displayed significant levels of Ca, Mg, Mn, and Zn. The seeds presented high essential fatty acids content, such as linoleic, palmitic, and oleic acids. Seed and peel ethanolic extracts showed phenolic compounds correlated to their antioxidant and AChE inhibitory activities. Additionally, the seed ethanolic extract exhibited in vivo neuroprotective effect. In vivo studies are an important step to determine the real potential of phytochemicals since they overcome many problems inherent to in vitro models. Therefore, the positive in vivo results encourage research of this extract as a potential candidate to develop drugs to treat patients in the early stages of neurodegenerative illnesses such as Alzheimer’s and Parkinson’s diseases. The PSMS chemical profile of the residues led to the identification of fifty-five metabolites, including phenolic, hydroxycinnamic acids, flavonoids, and alkaloids, most of them already reported as pharmacologically active compounds. Quantification of the phytochemicals will be the object of future work. In addition, toxicity, bioavailability, and suitable formulations should be further investigated before using avocado residues in pharmacotherapy, but the current results pave the way to deeper studies on the in vivo potential of avocado residues as a bioresource in the development of low-cost drugs and functional foods with neuroprotective effects. 

## Figures and Tables

**Table 1:** Mineral content in avocado fresh peels and seeds.

Minerals (mg/100 g of Sample)	Peels ^1^	Seeds ^1^
Ca	26.78 ± 2.06	41.14 ± 8.50
Cu	0.20 ± 0.74	0.48 ± 0.01
Fe	0.72 ± 2.06	1.04 ± 7.40
Mg	23.87 ± 3.09	31.41 ± 1.82
Mn	4.23 ± 10.34	1.80 ± 6.48
Zn	0.67 ± 7.02	1.11 ± 0.57

^1^ Values represent mean standard deviations (*n* = 5).

**Table 2:** Fatty acids composition of hexane and ethanolic extracts of avocado peels and seeds.

	Fatty Acids Contents (%)			
Fatty Acids	Peels		Seeds	
	PEL-H	PEL-ET	SED-H	SED-ET
Miristic 14:0	0.7	1.7	0.7	1.5
Palmitic 16:0	42.5	47.9	23.0	22.2
Palmitoleic 16:1	2.7	1.8	2.9	3.2
Stearic 18:0	7.0	22.2	4.1	14.7
Oleic 18:1	18.2	2.5	17.3	16.2
Linoleic 18:2	4.5	0.7	34.8	27.4
Linolenic 18:3	1.0	0.4	3.0	1.6
Total saturated fatty acids	50.2	71.8	27.8	38.4
Total unsaturated fatty acids	26.4	5.4	58	48.4
Total	76.6	77.2	85.8	86.8

SED-H: seed hexane extract; SED-ET: seed ethanolic extract; PEL-H: peel hexane extract, PEL-ET: peel ethanolic extract.

**Table 3:** Concentrations of phenols and flavonoids in different extracts of avocado peels and seeds.

Assays	Extracts			
	PEL-H	PEL-ET	SED-H	SED-ET
Total phenolic content (TPC) *	26.33 ± 0.48 ^g^	35.40 ± 0.60 ^d^	32.48 ± 2.00 ^e^	32.15 ± 0.39 ^fe^
Total flavonoid content (TFC) **	1243.78 ± 32.33 ^j^	694.058 ±1.490 ^l^	1199.04 ± 49.39 ^k^	640.72 ± 9.30 ^l^

SED-H: seed hexane extract; SED-ET: seed ethanolic extract; PEL-H: peel hexane extract, PEL-ET: peel ethanolic extract. * (TPC) = expressed as mg gallic acid/g extract; ** (TFC) = expressed in mg quercetin/g of extract. Data are expressed as the mean ± SD of five replicates. Different letters in the columns indicate statistical difference according to Tukey’s test (*p* < 0.05).

**Table 4:** Total antioxidant capacity, antioxidant activity determined by DPPH radical scavenging and ferric reducing power, and acetylcholinesterase inhibition of avocado peels and seeds.

Assays	Extracts			
	PEL-H	PEL-ET	SED-H	SED-ET
Total antioxidant capacity *	26.33 ± 0.48 ^g^	35.40 ± 0.60 ^d^	32.48 ± 2.00 ^e^	32.15 ± 0.39 ^fe^
DPPH scavenging (%)	7.77 ± 1.44 ^o^	52.20 ± 1.05 ^m^	35.89 ± 1.59 ^n^	37.60 ± 1.67 ^n^
Ferric reducing power (%) **	4.81 ± 1.37 ^g^	1.11 ± 0.25 ^i^	4.07 ± 1.21 ^gh^	2.38 ± 0.24 ^hi^
AChE inhibition (%)	70.8 ± 9.7 ^rq^	85.6 ± 11.1 ^pq^	65.0 ± 8.9 ^s^	78.0 ± 6.8 ^qr^

SED-H: seed hexane extract; SED-ET: seed ethanolic extract; PEL-H: peel hexane extract, PEL-ET: peel ethanolic extract, DPPH: 2,2-Diphenyl-1-picrylhydrazyl. * (TPC) = expressed as mg gallic acid/g extract; ** (TFC) = expressed in mg quercetin/g of extract. Data are expressed as the mean ± SD of five replicates. Different letters in the columns indicate statistical differences according to Tukey’s test (*p* < 0.05). Controls: Ferric reducing power (ascorbic acid): 98.89 ± 9.36%; DPPH capture assay (ascorbic acid): 79.16 ± 0.37%; AChE inhibition (eserine): 91.5 ± 1.6%.

**Table 5:** Compounds tentatively identified in avocado seed and peel by (−) PSMS.

*m*/*z*	Compound	Chemical Structure	Class	MS/MS	Extract	Reference
151	Vanillin	C_8_H_8_O_3_	Aldehyde	136, 107, 93	S, P	[43]
179	Caffeic acid	C_9_H_8_O_4_	Phenolic acid	151, 135	S	[43]
191	Quinic acid	C_7_H_12_O_6_	Phenolic acid	93, 111	S, P	[43]
197	Syringic acid	C_9_H_10_O_5_	Phenolic acid	153, 141, 125	S	[43]
209	5-Hydroxyferulic acid	C_10_H_10_O_5_	Phenolic acid	191, 165, 118	S	[44]
223	Sinapic acid	C_11_H_12_O_5_	Phenolic acid	179, 151, 85	S	[43]
269	Apigenin	C_15_H_10_O_5_	Flavonoid	252, 223, 197	S, P	[43]
285	Kaempferol	C_15_H_10_O_6_	Flavonoid	255, 224, 213	S, P	[43]
289	Catechin	C_15_H_13_O_6_	Flavonoid	274, 245, 217, 199	S, P	[44]
301	Quercetin	C_15_H_10_O_7_	Flavonoid	272, 265, 123	S, P	[43]
315	Hydroxytyrosol hexoside	C_14_H_20_O_8_	Phenolic glycoside	297, 269, 243	S, P	[44]
325	p-coumaroyl hexose	C_15_H_17_O_8_	Phenolic glycoside	261, 197, 183, 170	S, P	[44]
337	3-O-p-coumaroylquinic acid	C_16_H_18_O_8_	Phenolic compound	293, 237, 183	S, P	[44]
341	Caffeic acid-hexoside	C_15_H_18_O_9_	Phenolic glycoside	280, 185, 183, 179	S, P	[44]
353	5-O-caffeoylquinic acid	C_16_H_18_O_9_	Phenolic compound	309, 211, 191, 183	S, P	[43]
417	Kaempferol-O-pentoside	C_20_H_18_O_10_	Flavonoid glycoside	404, 344, 289	S, P	[44]
419	Cyanidin 3-O-pentatoside	C_20_H_19_O_10_	Flavonoid glycoside	395, 361, 292, 287	S, P	[44]
431	Vitexin	C_21_H_20_O_10_	Flavonoid glycoside	362, 351, 311, 196	S	[44]
433	Peonidin 3-O-pentoside	C_21_H_21_O_11_	Flavonoid glycoside	420, 389, 301, 205	S, P	[44]
435	Phloridzin	C_21_H_24_O_10_	Flavonoid glycoside	426, 416, 369	S	[43]
447	Kaempferol-O-hexoside	C_21_H_19_O_11_	Flavonoid glycoside	420, 403, 352, 301	S, P	[44]
449	Dihydroquercetin-3,5-rhamnoside	C_21_H_22_O_11_	Flavonoid glycoside	430, 303, 298, 286	S	[44]
451	Cinchonain	C_24_H_20_O_9_	Flavonoid	424, 414, 377	S, P	[44]
461	Isorhamnetin-O-coumaroyl	C_22_H_22_O_11_	Flavonoid	461, 417, 216	S, P	[44]
463	Quercetin-3-hexoside	C_21_H_20_O_12_	Flavonoid glycoside	464, 384, 316, 300	S	[44]
473	Quercetin-3-O-hexoside	C_21_H_19_O_12_	Flavonoid glycoside	467, 436, 372	S, P	[45]
477	Quercetin glucuronide	C_21_H_18_O_13_	Flavonoid	431, 262, 231	S	[44]
487	Caffeoyl hexose-deoxyhexoside	C_22_H_31_O_12_	Flavonoid	442, 298, 173	S	[44]
491	Dimethyl ellagic acid hexoside	C_22_H_22_O_13_	Flavonoid	343, 275, 269	S, P	[44]
563	Apigenin-C-hexoside-C-pentoside	C_26_H_28_O_14_	Flavonoid glycoside	531, 446, 298	S	[44]
575	Procyanidin dimer A	C_30_H_24_O_12_	Flavonoid	431, 404, 329	S	[43]
577	Procyanidin dimer B	C_30_H_25_O_12_	Flavonoid	532, 516, 420	S	[44]
579	Luteolin 7-O-(2”-O-pentosyl)hexoside	C_26_H_28_O_15_	Flavonoid glycoside	560, 542, 514	S, P	[44]
593	Catechin dihexoside	C_27_H_29_O_15_	Flavonoid glycoside	574, 495, 347	S	[44]
595	Quercetin-O-pentatosyl-hexoside	C_26_H_28_O_16_	Flavonoid glycoside	562, 558, 497	S, P	[44]
609	Rutin	C_27_H_30_O_16_	Flavonoid glycoside	573, 564, 208	S, P	[44]
625	Quercetin-3,4’-O-diglucoside	C_27_H_30_O_17_	Flavonoid glycoside	605, 588, 581	S, P	[43]
863	Procyanidin trimer A	C_45_H_36_O_18_	Flavonoid	845, 826, 555	S, P	[43]
865	Procyanidin trimer B-isomer 1	C_45_H_38_O_18_	Flavonoid	829, 735, 560	S	[43]

S: Avocado seed; P: Avocado peel.

**Table 6:** Compounds tentatively identified in avocado seed and peel by (+) PSMS.

*m*/*z*	Compound	Chemical Structure	MS/MS	Extract	Reference
204	Anibine (**1**)	C_11_H_9_NO_3_	183, 188, 192	S	[48]
244	Duckeine (**2**)	C_13_H_11_NO_4_	226, 235, 187	S	[48]
256	Riparin I (**3**)	C_16_H_17_NO_2_	241, 187, 212	S, P	[48]
270	Norcanelilline (**6**)	C_17_H_19_NO_3_	214, 261, 240	S, P	[48]
272	Riparin II (**4**)	C_16_H_17_NO_3_	263, 250, 254	S, P	[48]
282	Anicanine (**8**)	C_19_H_23_NO_3_	200, 183, 192	S	[48]
288	Riparin III (**5**)	C_16_H_17_NO_4_	270, 106, 271	S, P	[48]
296	(-)-α-8-methyl-pseudoanibacanine (**9**)	C_19_H_21_NO_3_	279, 287, 239	S, P	[48]
300	N-methylcoclaurine (**7**)	C_18_H_21_NO_3_	283, 291, 227	S, P	[48]
305	Ceceline (**10**)	C_19_H_16_N_2_O_2_	263, 273, 287	S, P	[48]
312	(+)-Manibacanine (**11**)	C_19_H_21_NO_3_	116, 291, 243	S, P	[48]
325	Cassythicine (**12**)	C_19_H_19_NO_4_	287, 316, 307	S, P	[45]
328	Isoboldine (**13**)	C_19_H_21_NO_4_	297, 178, 310	S, P	[48]
330	Reticuline (**14**)	C_19_H_23_NO_4_	187, 218, 235	S, P	[48]
339	Nantenine (**15**)	C_20_H_21_NO_4_	249, 330, 321	S, P	[45]
424	Anibamine (**16**)	C_30_H_50_N^+^	334, 379, 418	S, P	[48]

## Supplementary Materials

The following supporting information can be downloaded at: https://www.mdpi.com/article/10.3390/molecules27061892/s1, Section S1: PSMS of the seed and ethanolic extracts and of the compounds identified, Figure S1: PS(+)MS full scan of ethanol extract from peel of avocado, Figure S2: Anibine PS(+)MS fragmentation and chemical structure, Figure S3: Duckeine PS(+)MS fragmentation and chemical structure, Figure S4: Riparin I PS(+)MS fragmentation and chemical structure, Figure S5: Norcanelilline PS(+)MS fragmentation and chemical structure, Figure S6: Riparin II PS(+)MS fragmentation and chemical structure, Figure S7: Anicanine PS(+)MS fragmentation and chemical structure, Figure S8: Riparin III PS(+)MS fragmentation and chemical structure, Figure S9: (-)-α-methylpseudoanibacanine PS(+)MS fragmentation and chemical structure, Figure S10: N- methylcoclaurine PS(+)MS fragmentation and chemical structure, Figure S11: Ceceline PS(+)MS fragmentation and chemical structure, Figure S12: (+)-manibacanine PS(+)MS fragmentation and chemical structure, Figure S13: Cassythicine PS(+)MS fragmentation and chemical structure, Figure S14: Isoboldine PS(+)MS fragmentation and chemical structure, Figure S15: Reticuline PS(+)MS fragmentation and chemical structure, Figure S16: Anibamine PS(+)MS fragmentation and chemical structure, Figure S17: PS(−)MS full scan of peel ethanolic extract of avocado, Figure S18: Vanillin PS(−)MS fragmentation and chemical structure, Figure S19: Caffeic acid PS(−)MS fragmentation and chemical structure, Figure S20: Quinic acid PS(−)MS fragmentation and chemical structure, Figure S21: Syrinc acid PS(−)MS fragmentation and chemical structure, Figure S22: 5-hydroxyferulic acid PS(−)MS fragmentation and chemical structure, Figure S23: Sinapic acid PS(−)MS fragmentation and chemical structure, Figure S24: Apigenin PS(−)MS fragmentation and chemical structure, Figure S25: Kaempferol PS(−)MS fragmentation and chemical structure, Figure S26: Catechin PS(−)MS fragmentation and chemical structure, Figure S27: Quercetin PS(−)MS fragmentation and chemical structure, Figure S28: Hydroxytyrosol glucoside PS(−)MS fragmentation and chemical structure, Figure S29: p-coumaroyl hexose PS(−)MS fragmentation and chemical structure, Figure S30: 3-O-p-coumaroylquinic acid PS(−)MS fragmentation and chemical structure, Figure S31: Caffeic acid hexoside PS(−)MS fragmentation and chemical structure, Figure S32: 5-O-caffeoylquinic acid PS(−)MS fragmentation and chemical structure, Figure S33: Kaempferol-O-pentoside PS(−)MS fragmentation and chemical structure, Figure S34: Cyanidin-3-O-arabinoside PS(−)MS fragmentation and chemical structure, Figure S35: Vitexin PS(−)MS fragmentation and chemical structure, Figure S36: Peonidin-3-O-pentoside PS(−)MS fragmentation and chemical structure, Figure S37: Phloridzin PS(−)MS fragmentation and chemical structure, Figure S38: Kaempferol-O-hexoside PS(−)MS fragmentation and chemical structure, Figure S39: Dihydroquercetin-3,5-rhamnoside PS(−)MS fragmentation and chemical structure, Figure S40: Isorhamnetin-O-coumaroyl PS(−)MS fragmentation and chemical structure, Figure S41: Quercetin-3-glucoside PS(−)MS fragmentation and chemical structure, Figure S42: Quercetin glucuronide PS(−)MS fragmentation and chemical structure, Figure S43: Caffeoyl hexose-deohyhexoside PS(−)MS fragmentation and chemical structure, Figure S44: Dimethyl ellagic acid hexoside PS(−)MS fragmentation and chemical structure, Figure S45: Apigenin-C-hexoside-C-pentoside PS(−)MS fragmentation and chemical structure, Figure S46: Luteolin-7-O-(2”-O-pentosyl)-hexoside PS(−)MS fragmentation and chemical structure, Figure S47: Catechin diglucopyranoside PS(−)MS fragmentation and chemical structure, Figure S48: Quercetin-3,4′-O-diglucoside PS(−)MS fragmentation and chemical structure.

## Sample Availability

Samples of the compounds are not available.
