# Supplementary material for: Phytochemicals of Avocado Residues as Potential Acetylcholinesterase Inhibitors, Antioxidants, and Neuroprotective Agents
Source: Molecules. 2022 Mar 15;27(6):1892. doi: 10.3390/molecules27061892 (PMC8953789; doi:10.3390/molecules27061892)

Supplementary file

Figure S1. PS(+)MS full scan of ethanol extract from peel of avocado

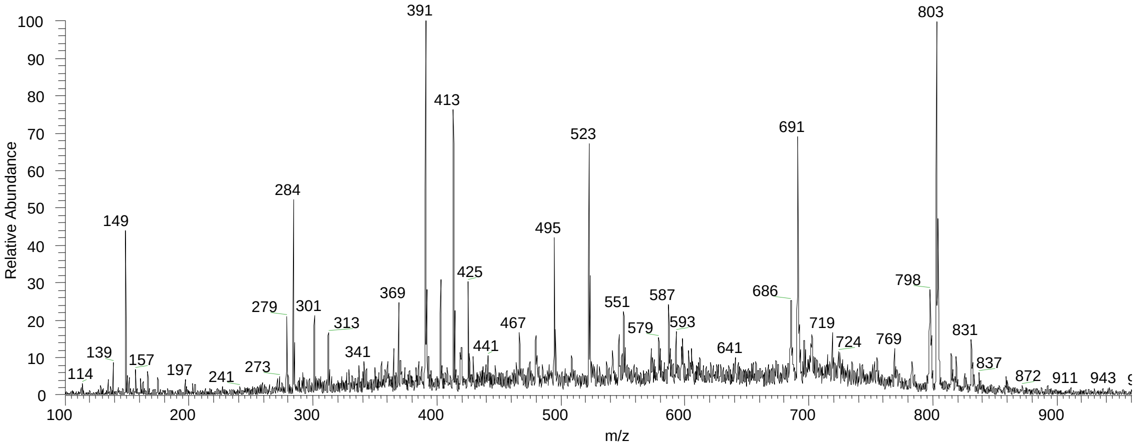

Figure S2. Anibine PS(+)MS fragmentation and chemical structure.

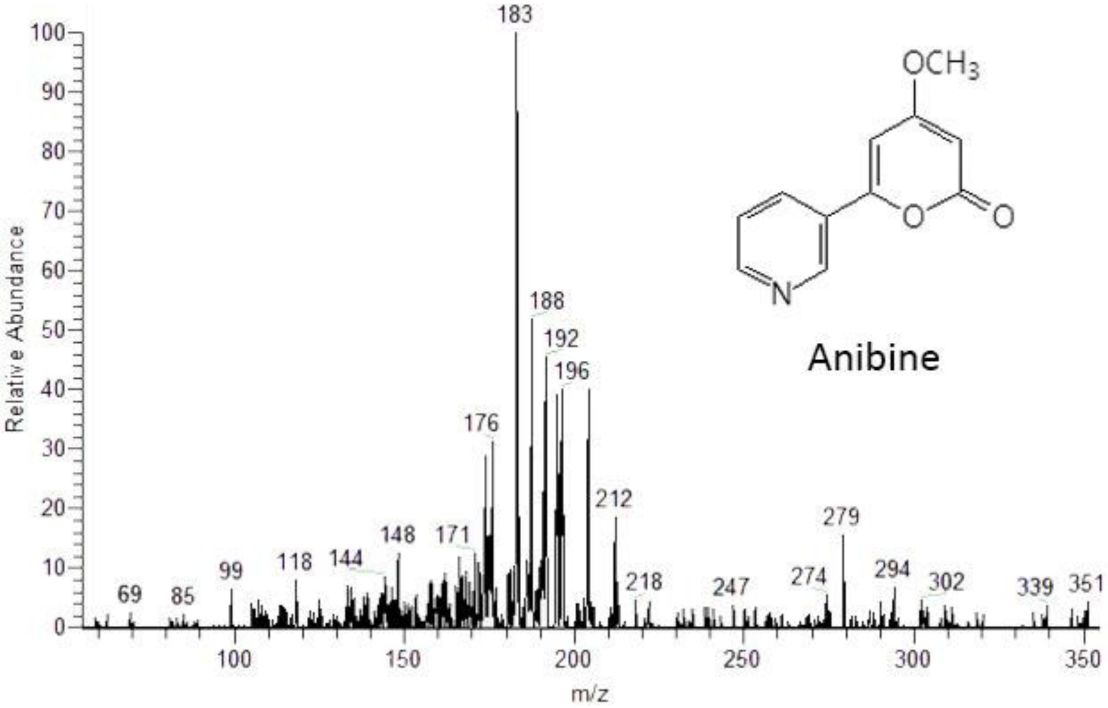

Figure S3. Duckeine PS(+)MS fragmentation and chemical structure.

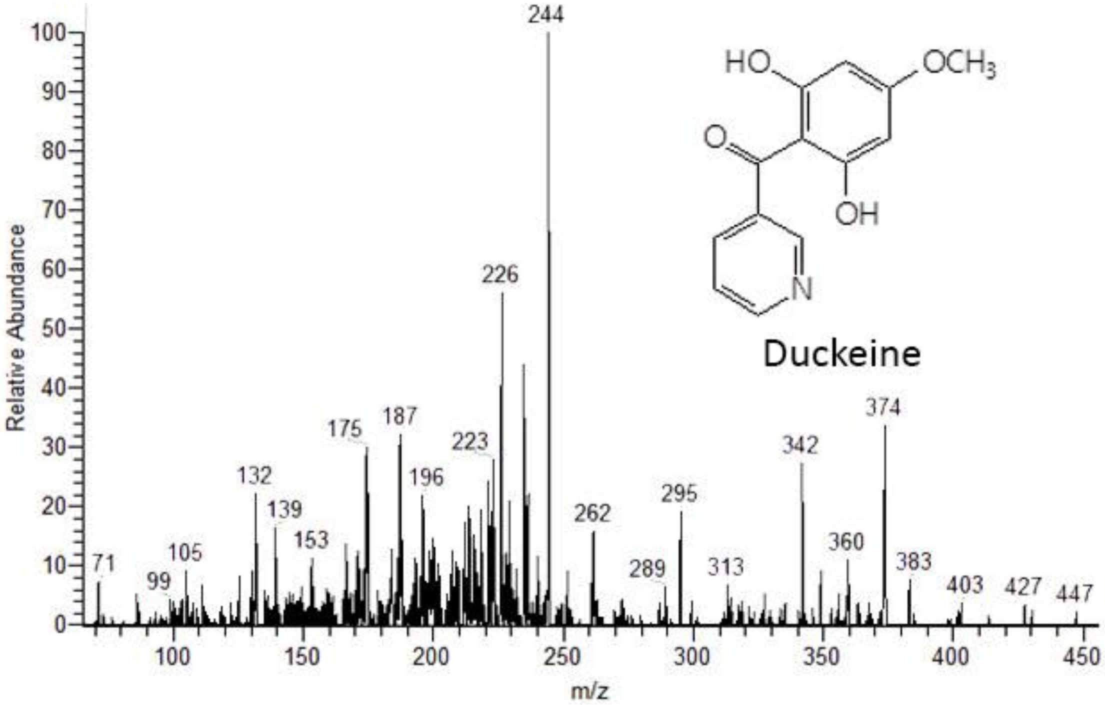

Figure S4. Riparin I PS(+)MS fragmentation and chemical structure.

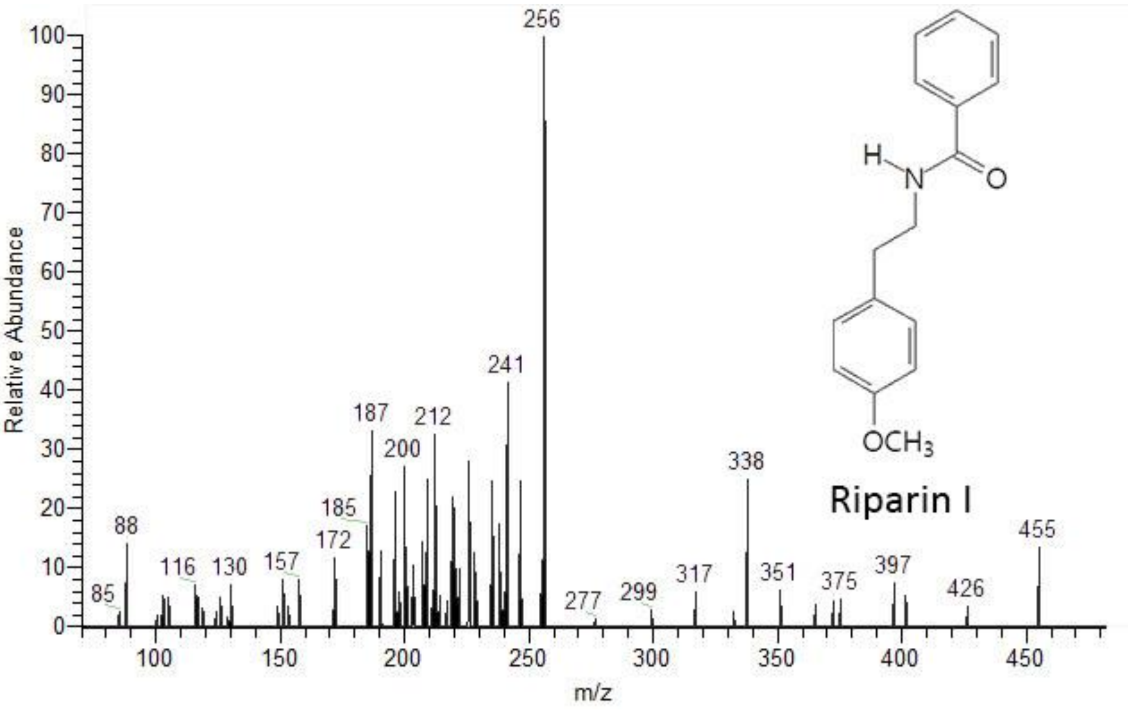

Figure S5. Norcanelilline PS(+)MS fragmentation and chemical structure.

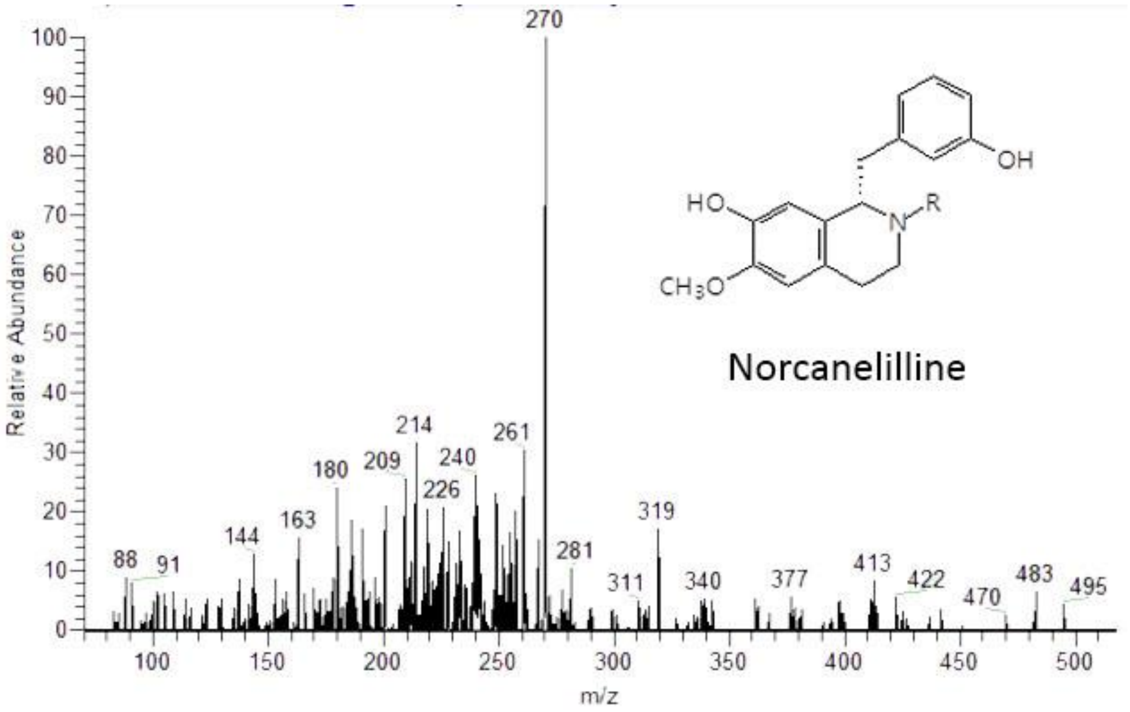

Figure S6. Riparin II PS(+)MS fragmentation and chemical structure.

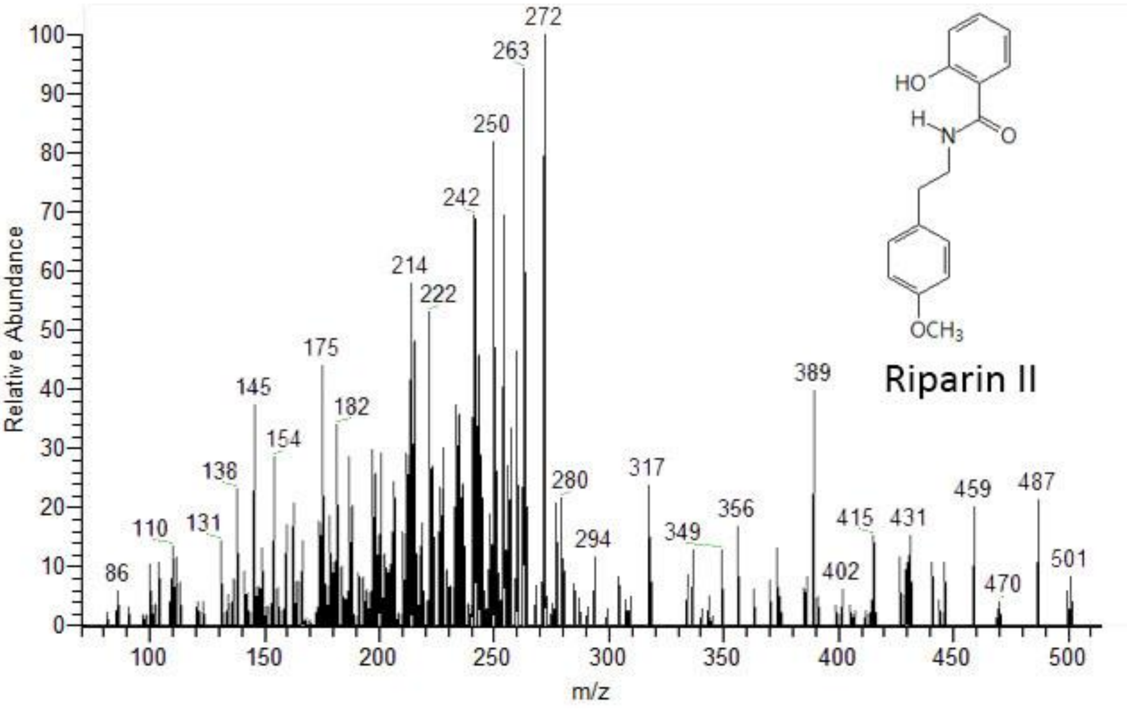

Figure S7. Anicanine PS(+)MS fragmentation and chemical structure.

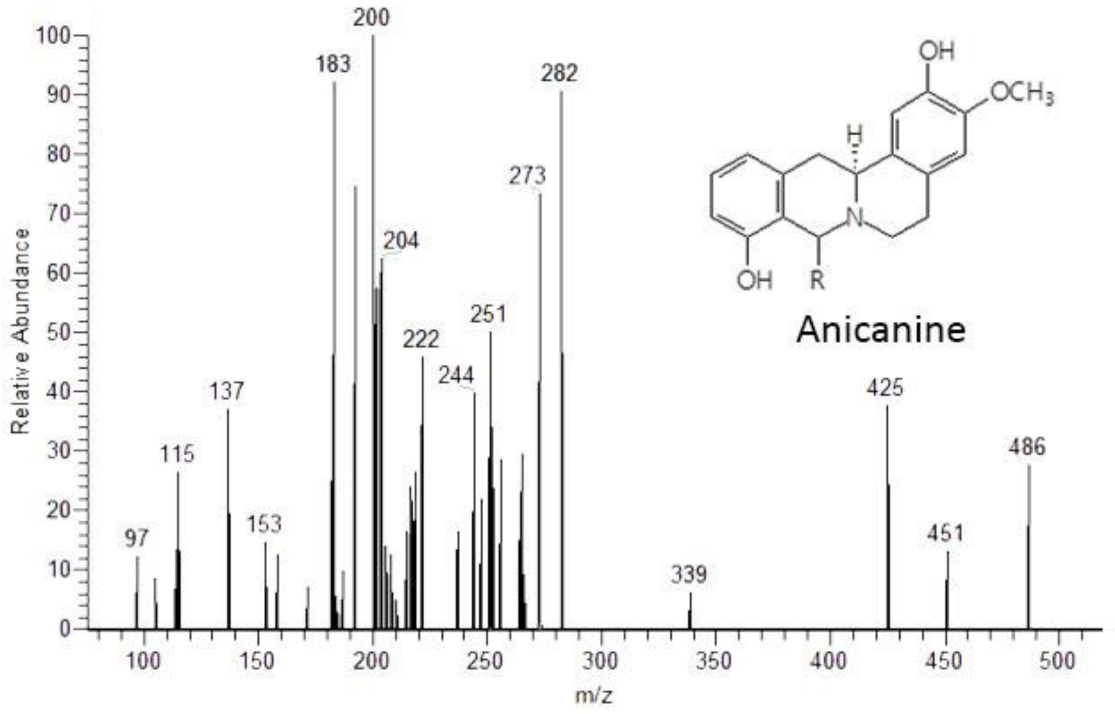

Figure S8. Riparin III PS(+)MS fragmentation and chemical structure.

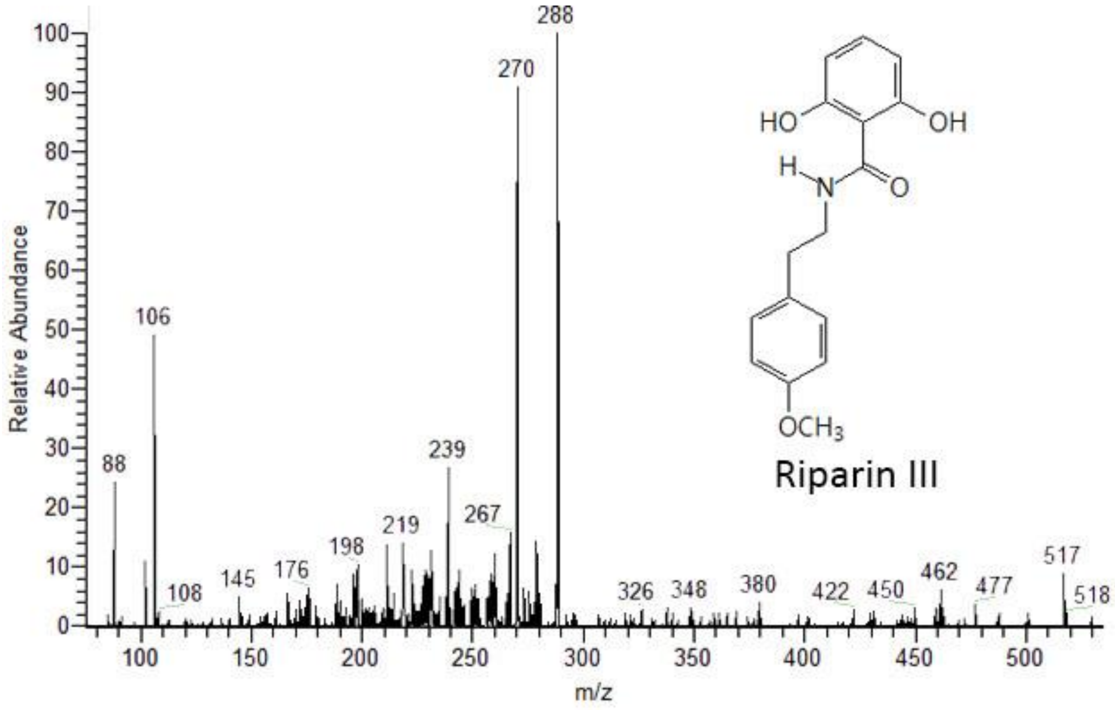

Figure S9. (-)-α-methylpseudoanibacanine PS(+)MS fragmentation and chemical structure.

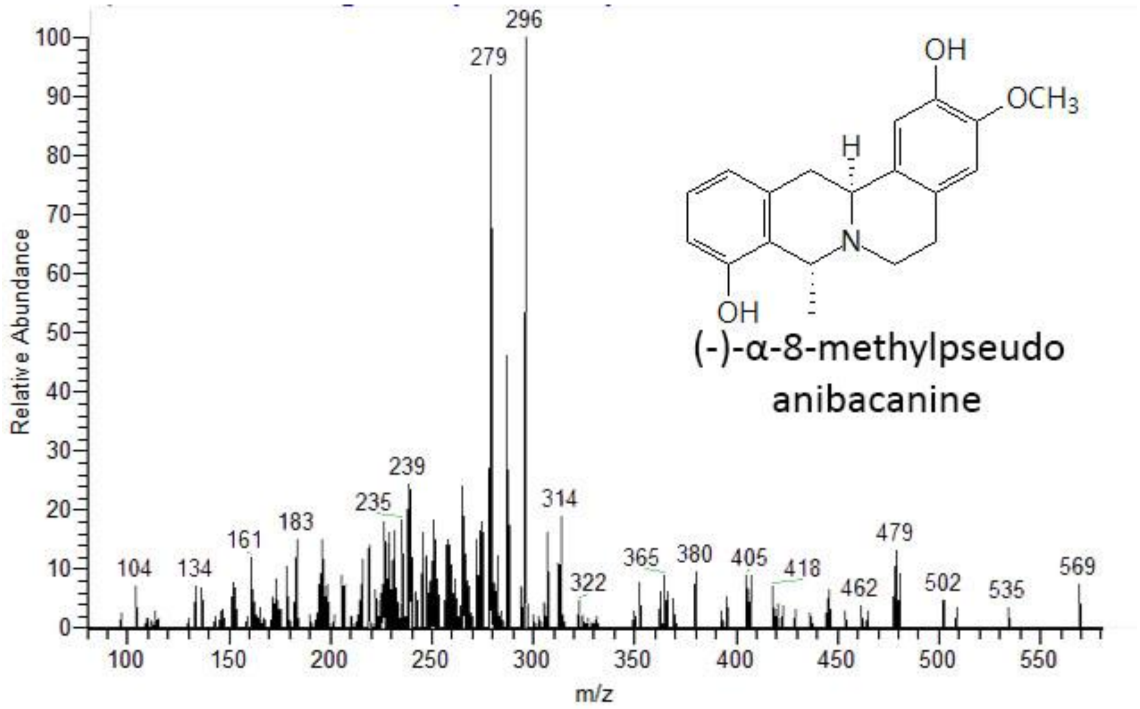

Figure S10. N- methylcoclaurine PS(+)MS fragmentation and chemical structure.

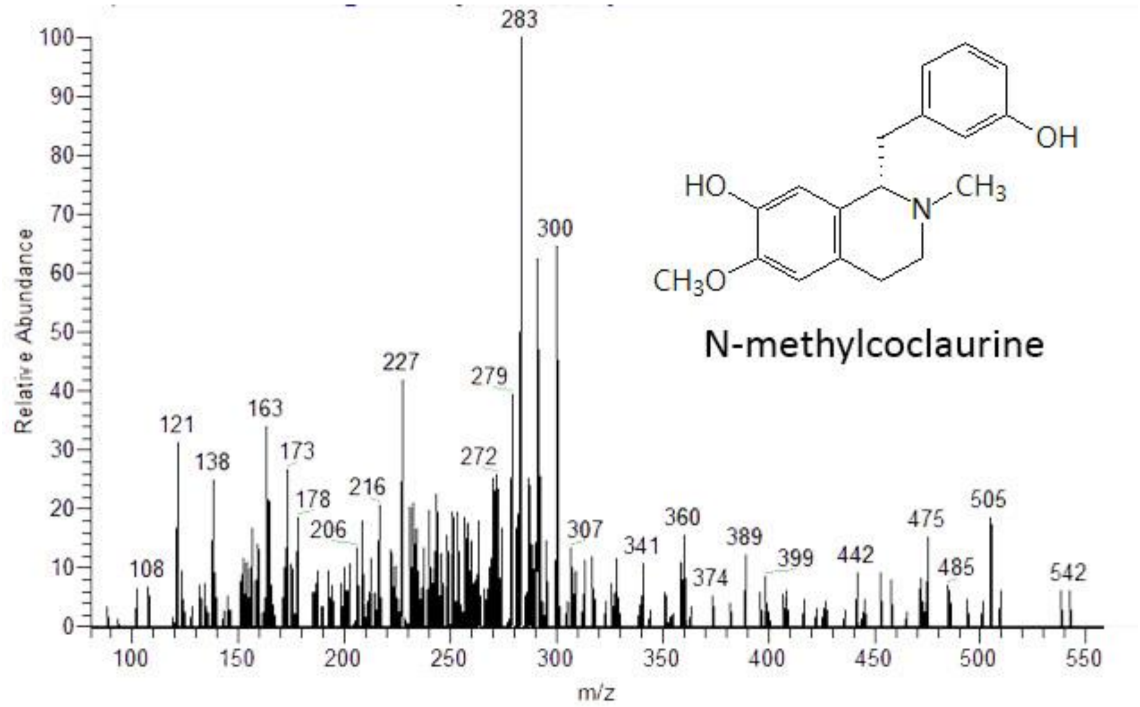

Figure S11. Ceceline PS(+)MS fragmentation and chemical structure.

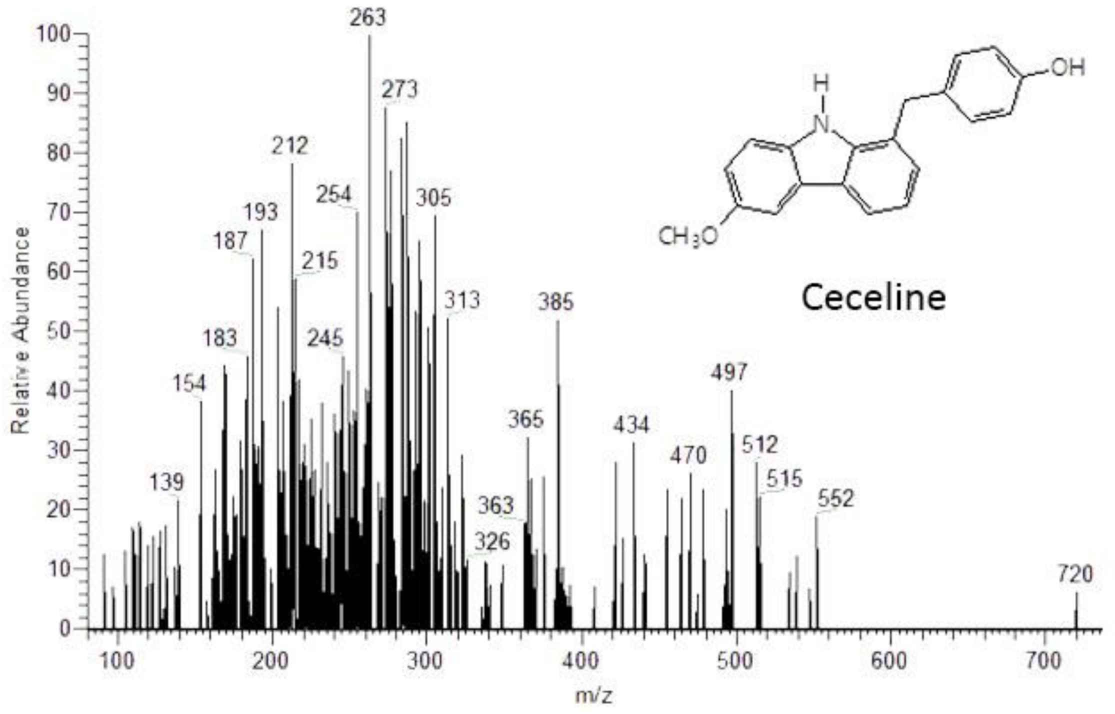

Figure S12. (+)-manibacanine PS(+)MS fragmentation and chemical structure.

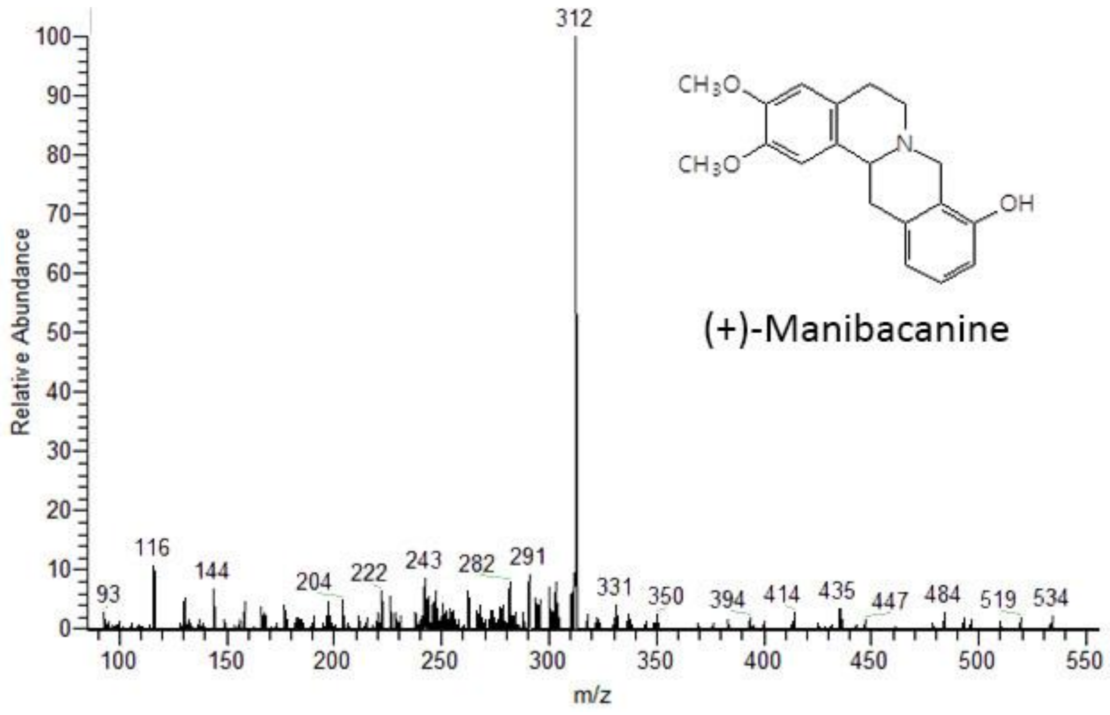

Figure S13. Cassythicine PS(+)MS fragmentation and chemical structure.

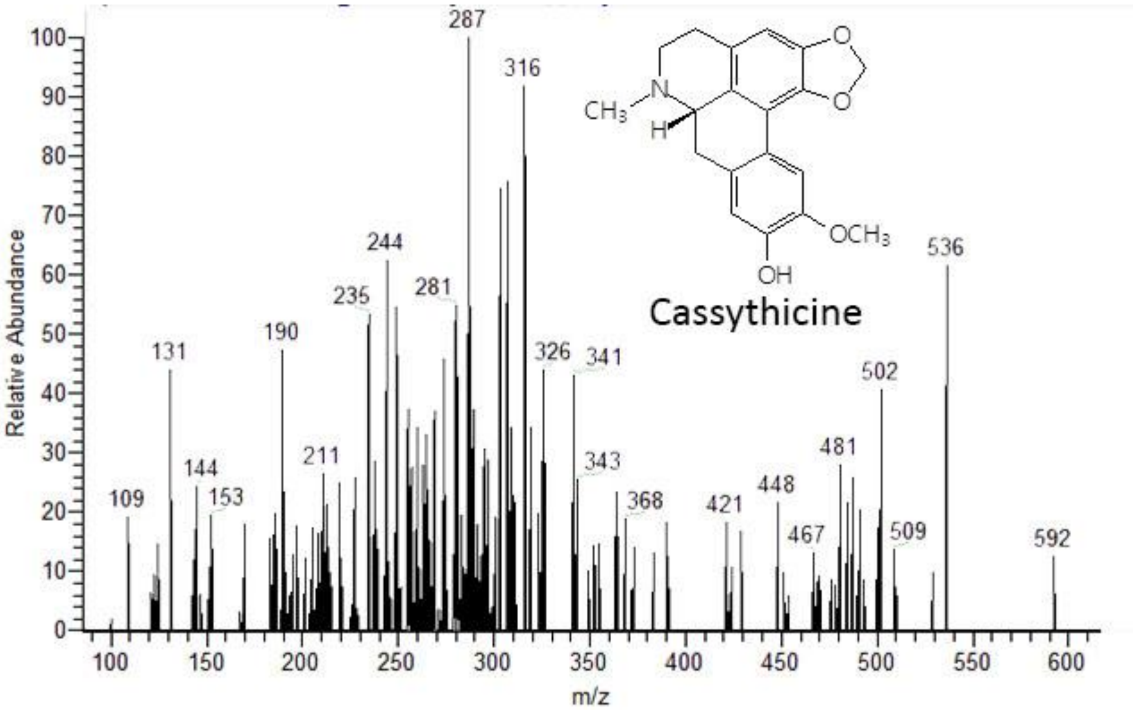

Figure S14. Isoboldine PS(+)MS fragmentation and chemical structure.

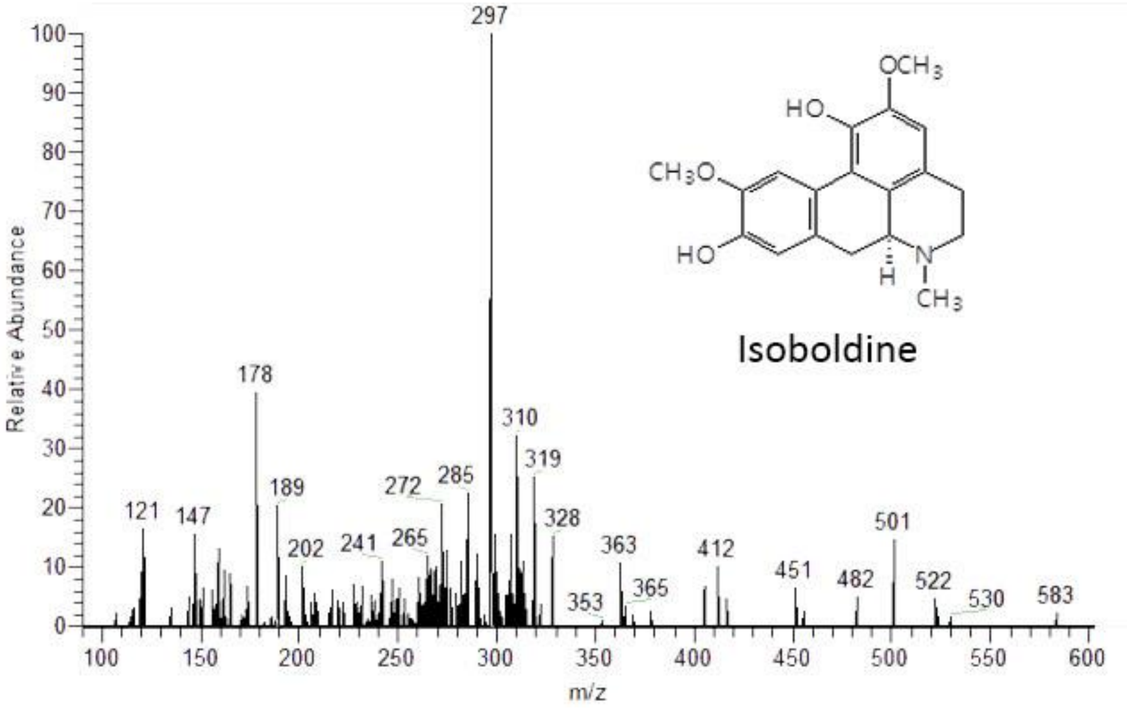

Figure S15. Reticuline PS(+)MS fragmentation and chemical structure.

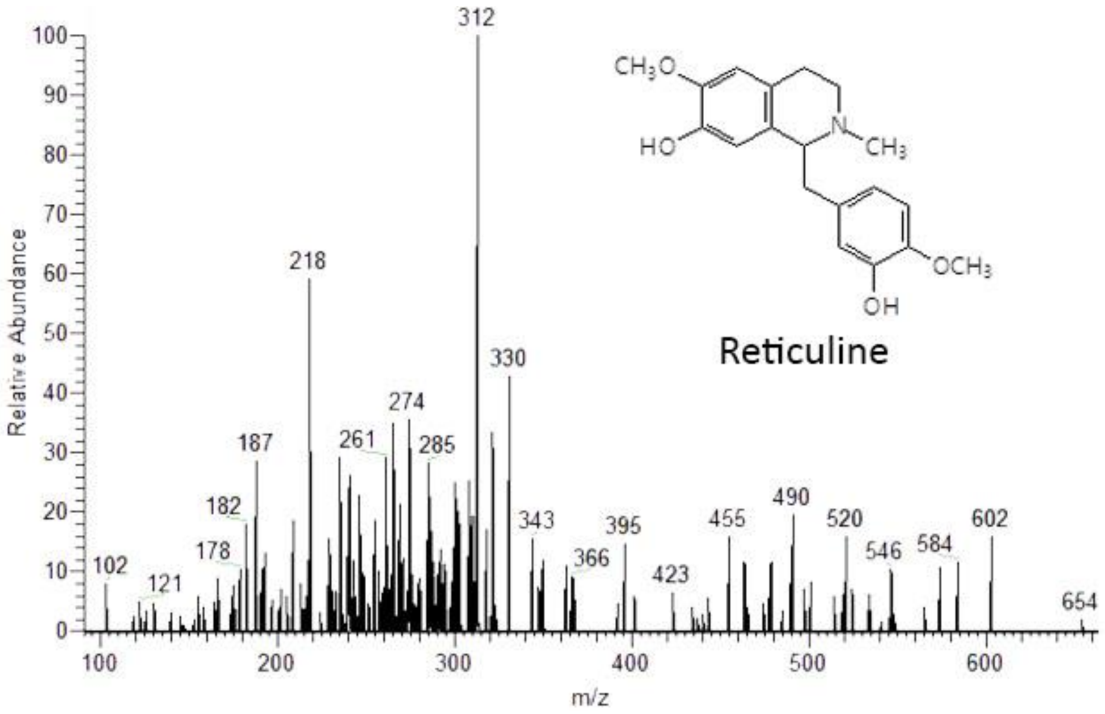

Figure S16. Anibamine PS(+)MS fragmentation and chemical structure.

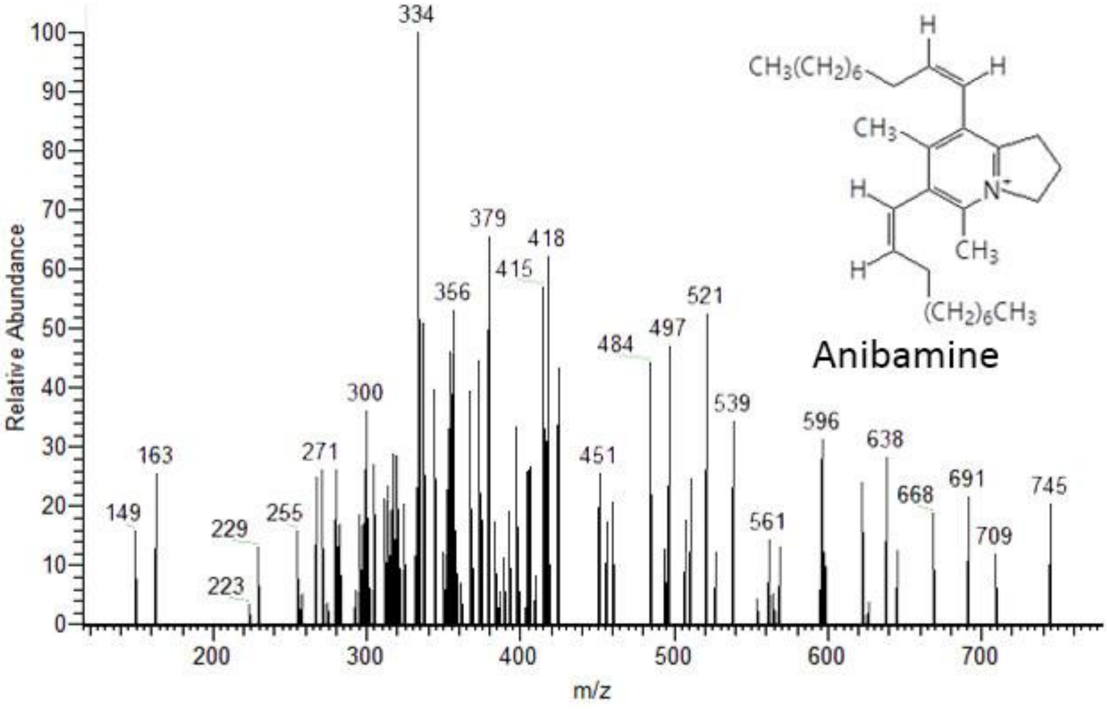

Figure S17. PS(+)MS full scan of ethanol extract from peel of avocado

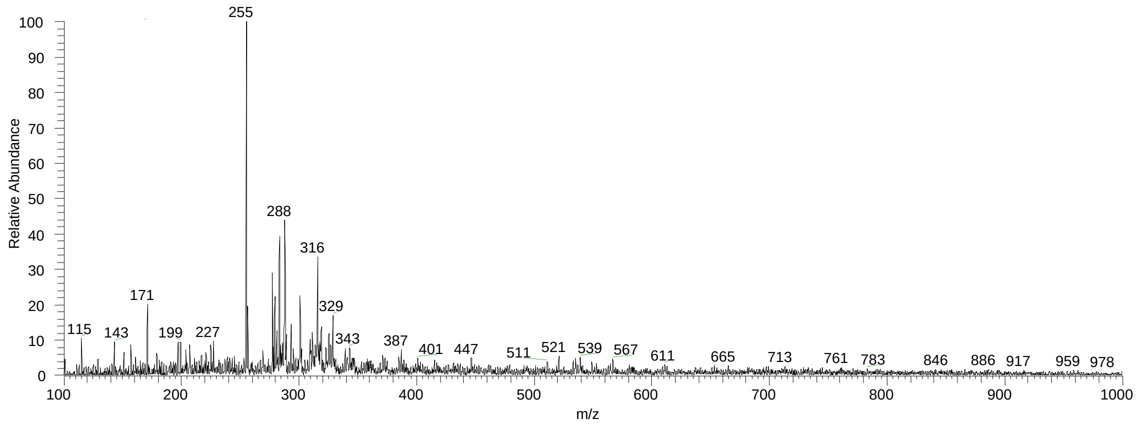

Figure S18. Vanillin PS(-)MS fragmentation and chemical structure.

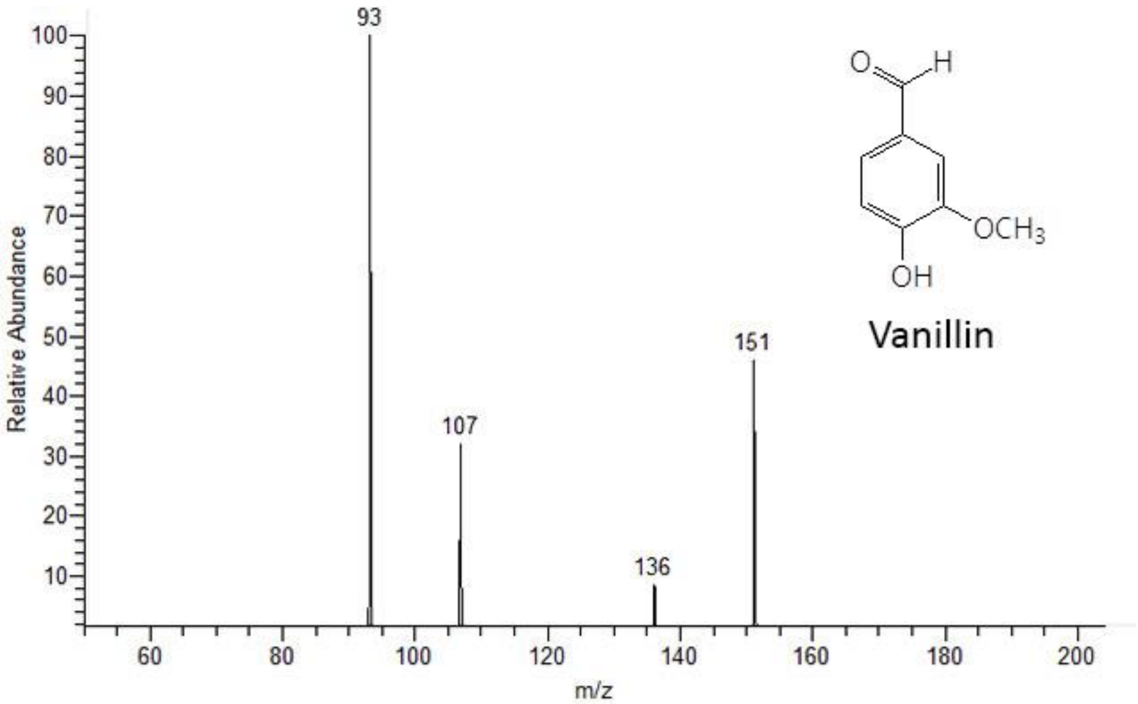

Figure S19. Caffeic acid PS(-)MS fragmentation and chemical structure.

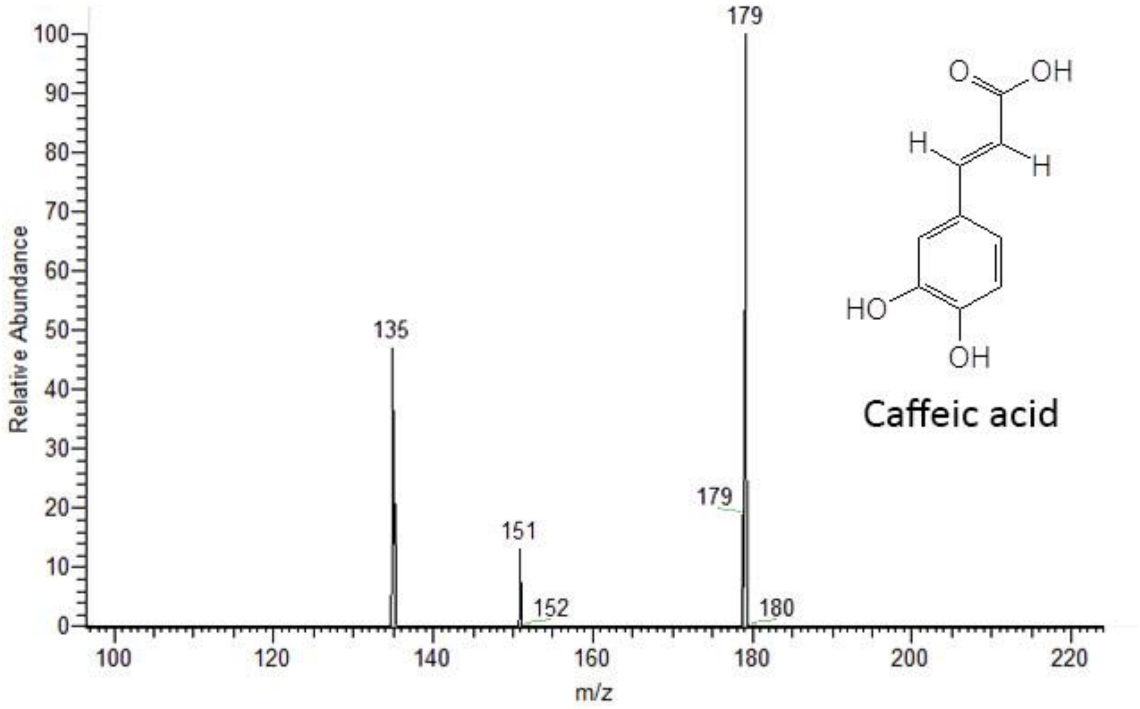

Figure S20. Quinic acid PS(-)MS fragmentation and chemical structure.

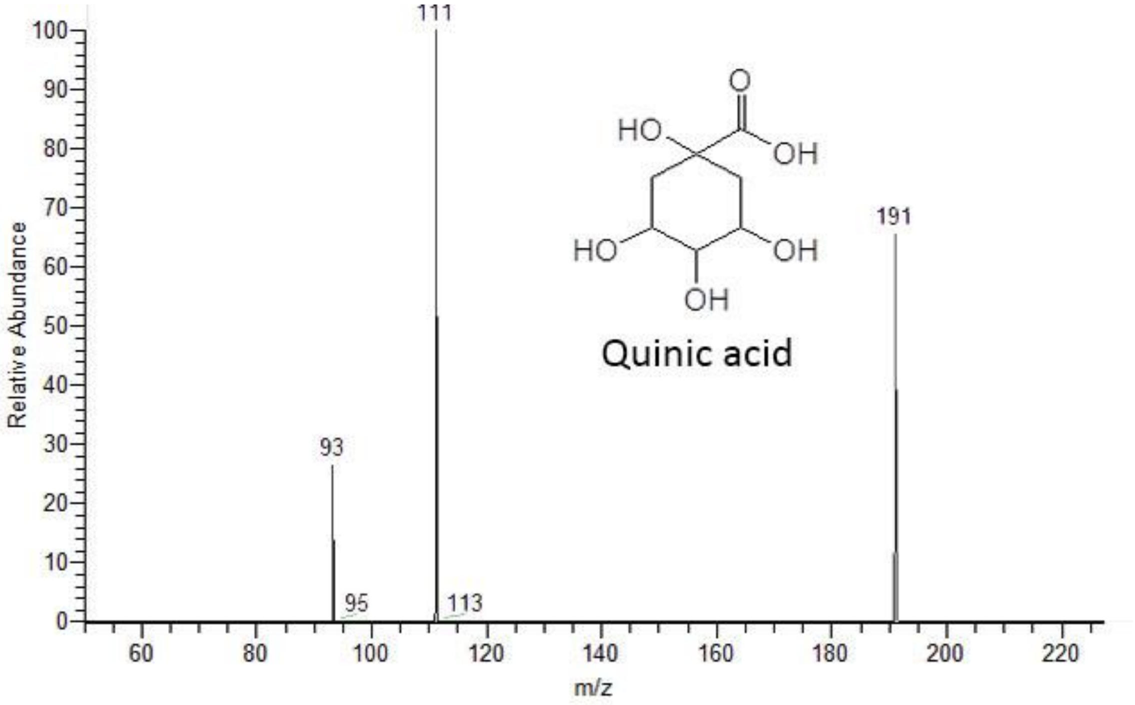

Figure S21. Syrinc acid PS(-)MS fragmentation and chemical structure.

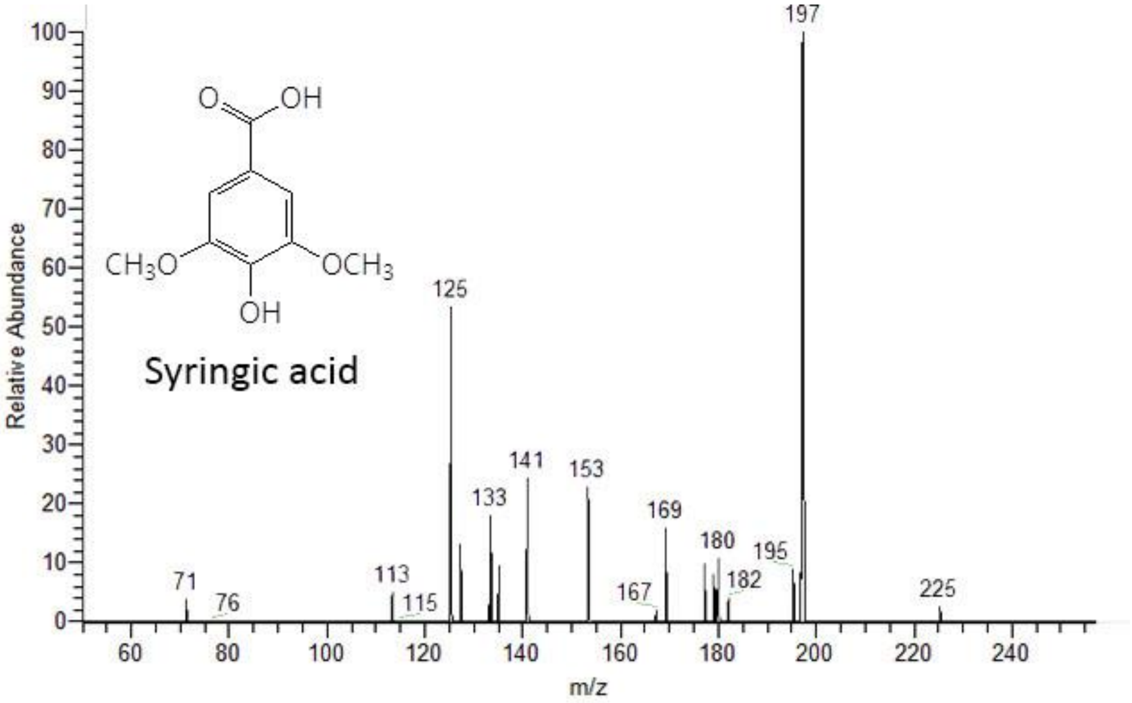

Figure S22. 5-hydroxyferulic acid PS(-)MS fragmentation and chemical structure.

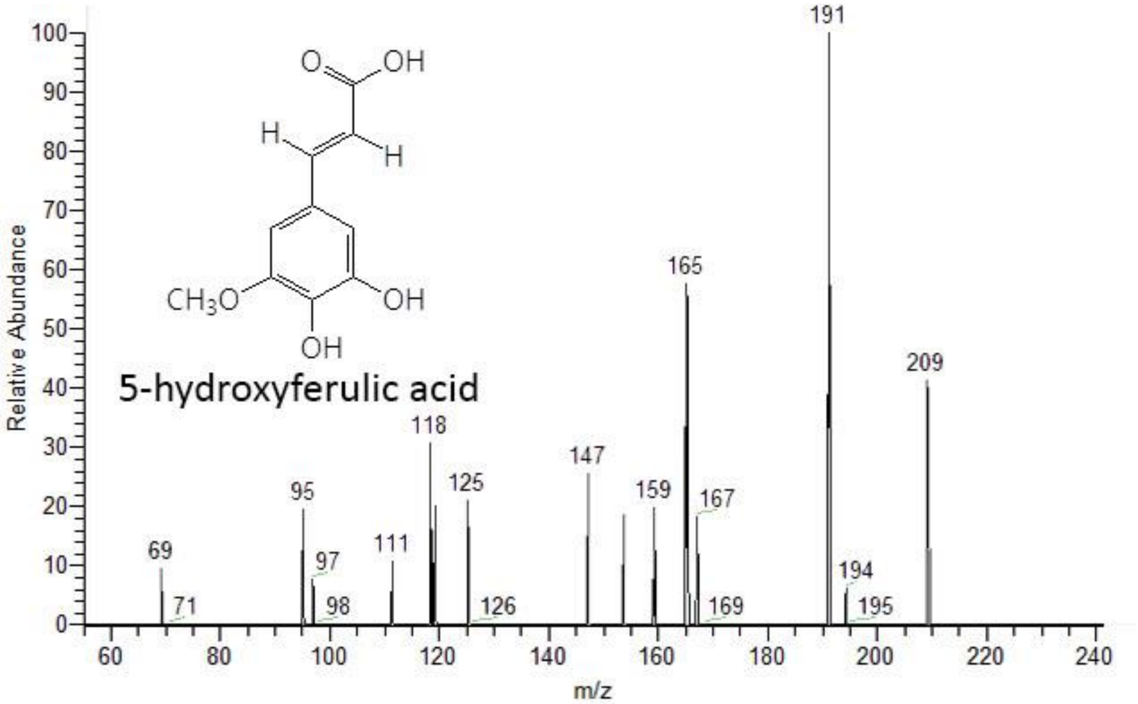

Figure S23. Sinapic acid PS(-)MS fragmentation and chemical structure.

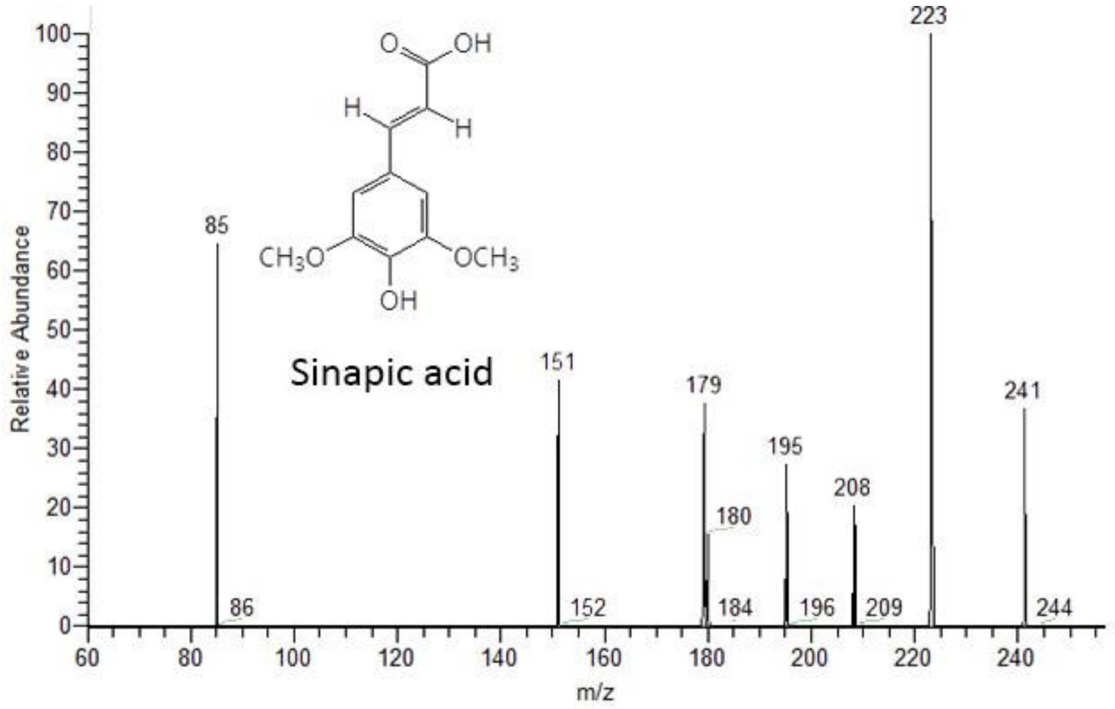

Figure S24. Apigenin PS(-)MS fragmentation and chemical structure.

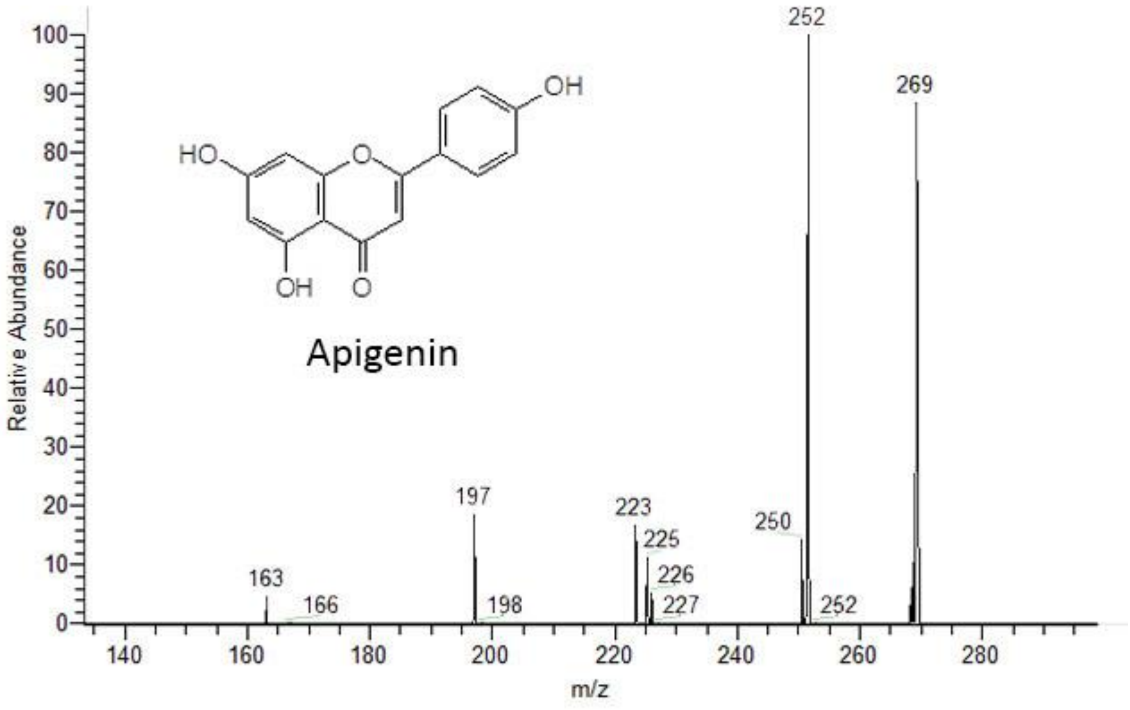

Figure S25. Kaempferol PS(-)MS fragmentation and chemical structure.

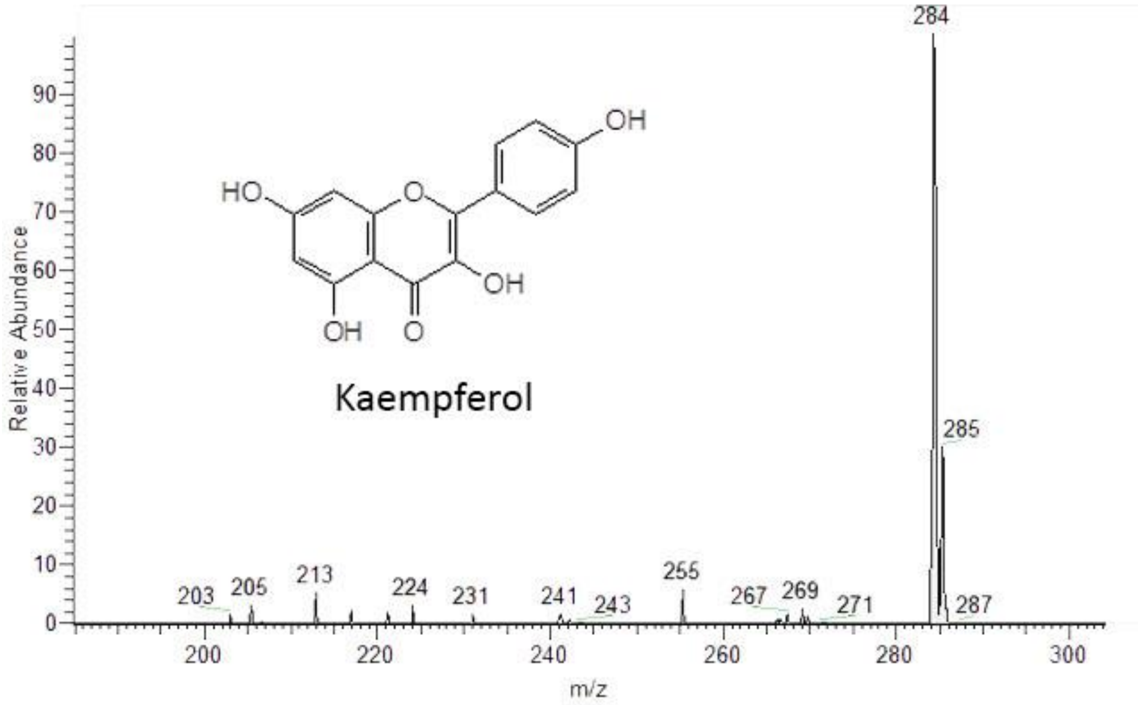

Figure S26. Catechin PS(-)MS fragmentation and chemical structure.

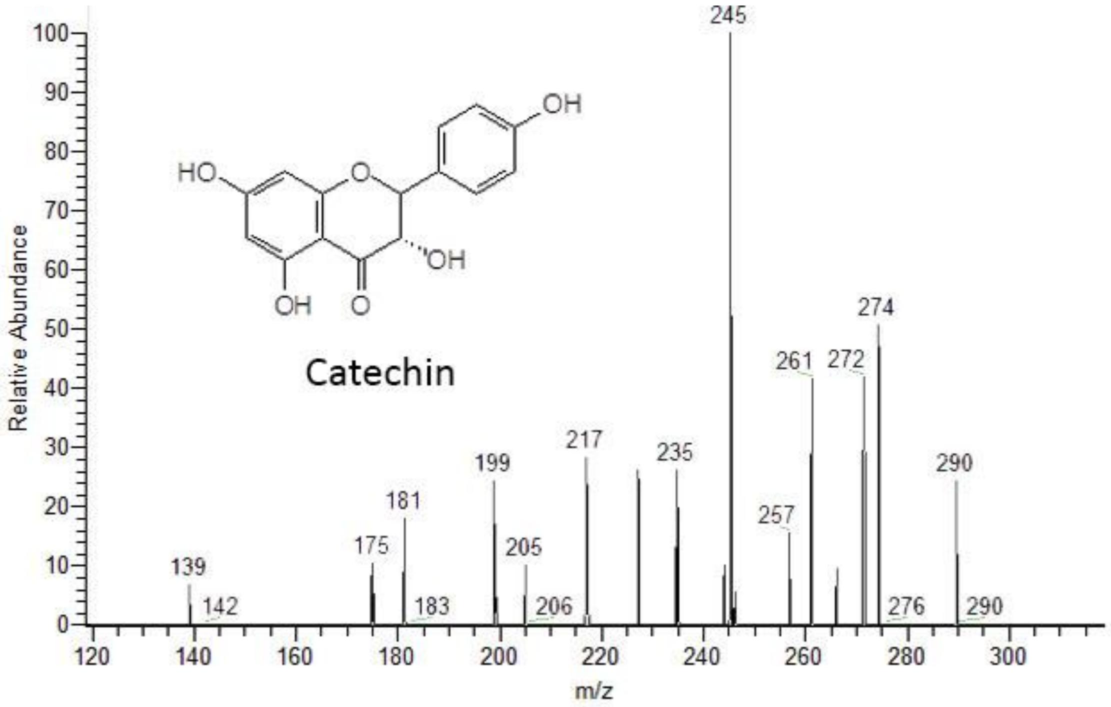

Figure S27. Quercetin PS(-)MS fragmentation and chemical structure.

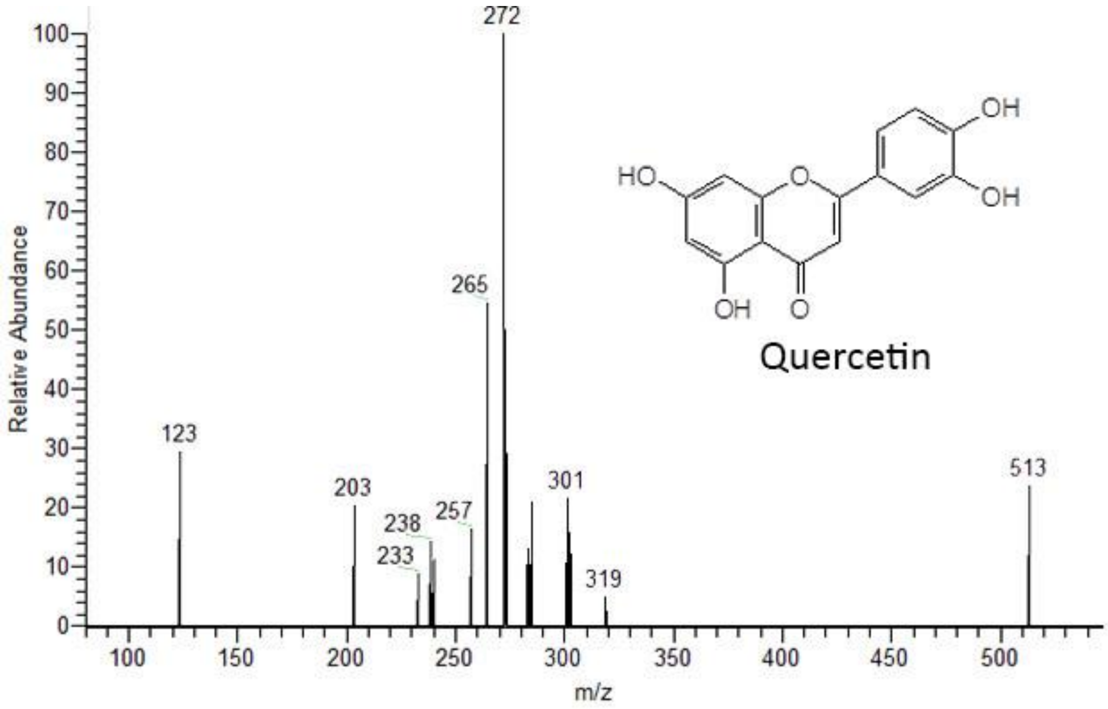

Figure S28. Hydroxytyrosol glucoside PS(-)MS fragmentation and chemical structure.

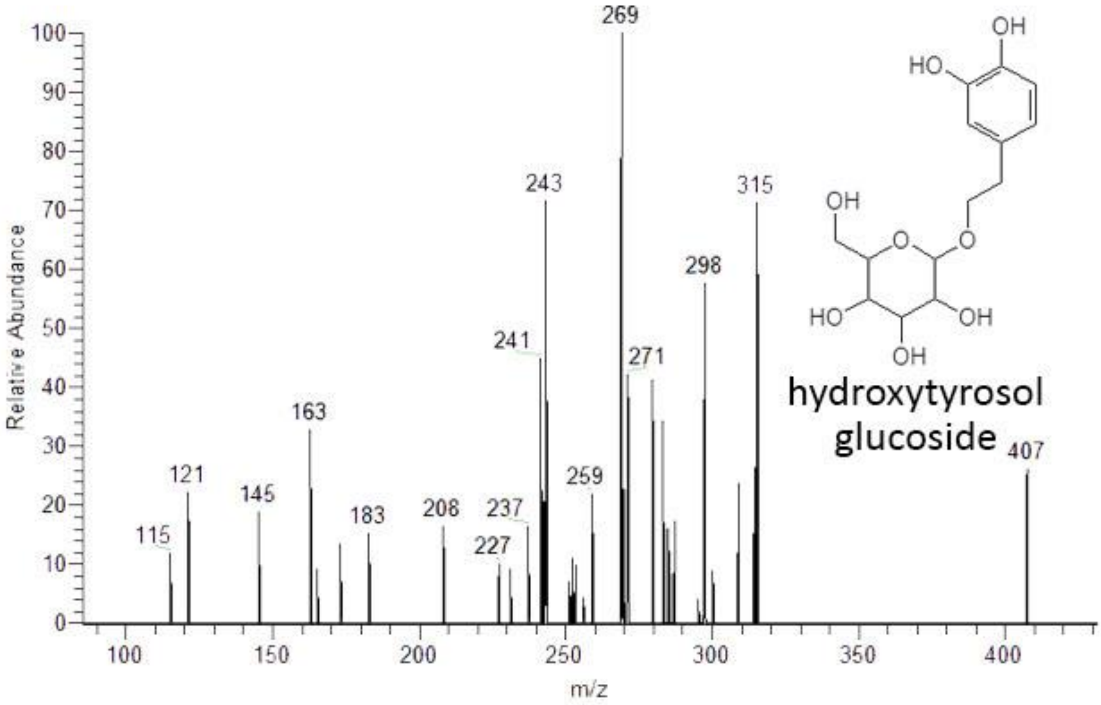

Figure S29. p-Coumaroyl hexose PS(-)MS fragmentation and chemical structure.

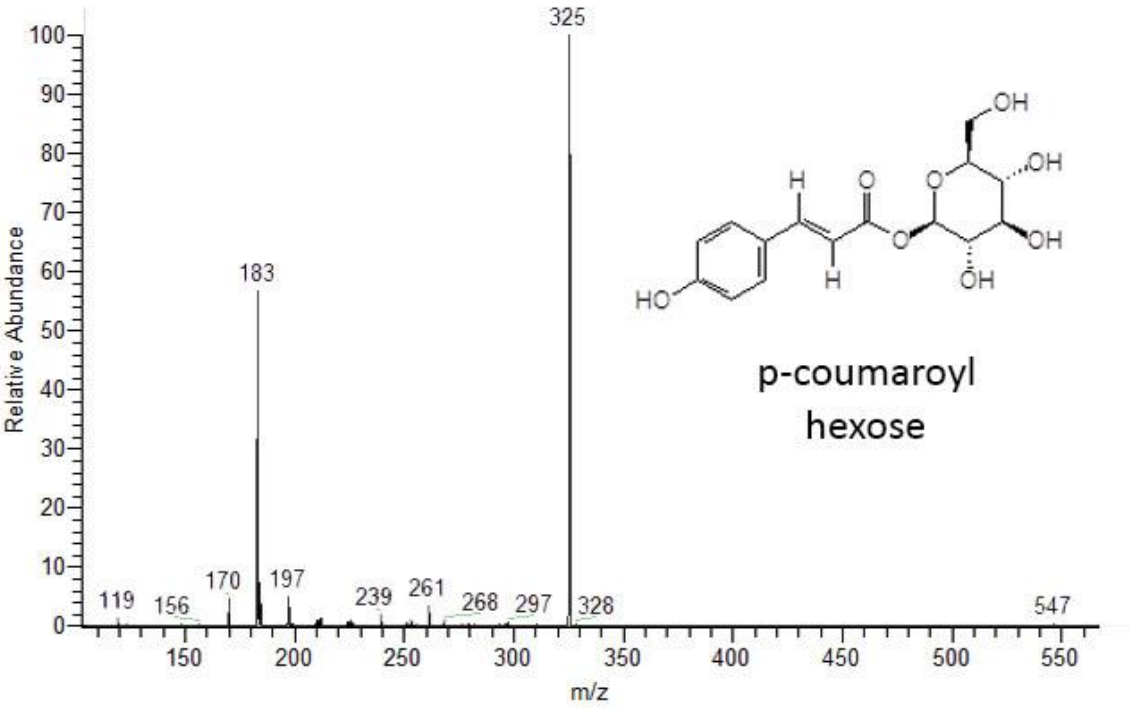

Figure S30. 3-O-p-coumaroylquinic acid PS(-)MS fragmentation and chemical structure.

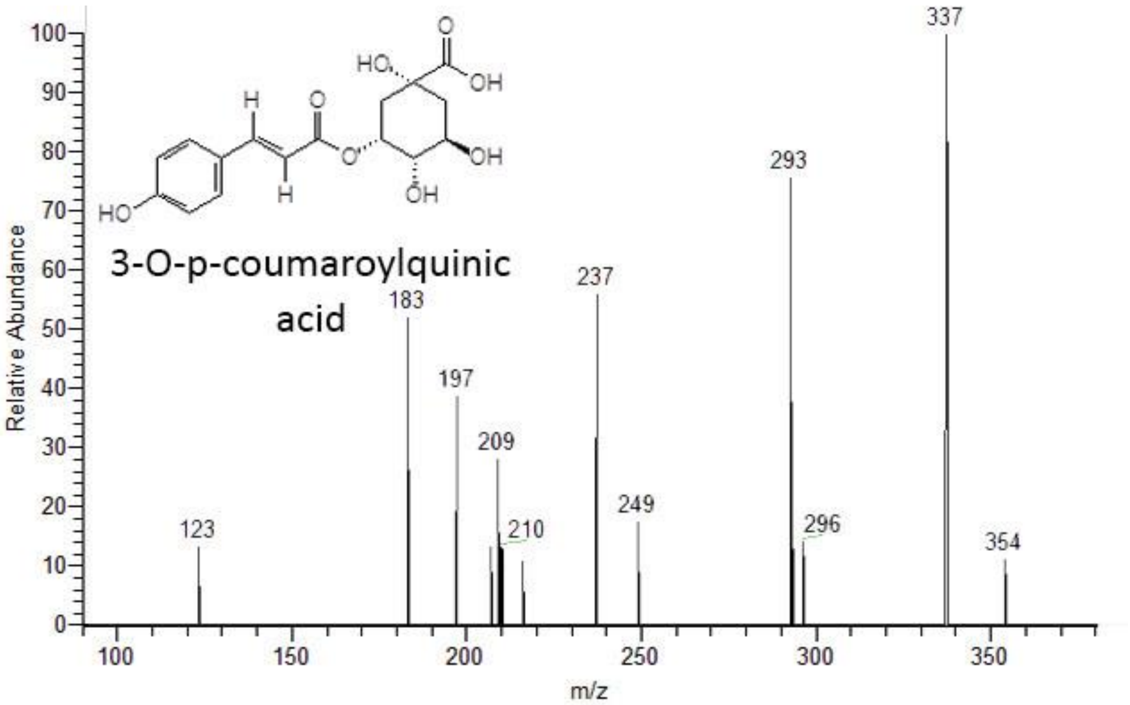

Figure S31. Caffeic acid hexoside PS(-)MS fragmentation and chemical structure.

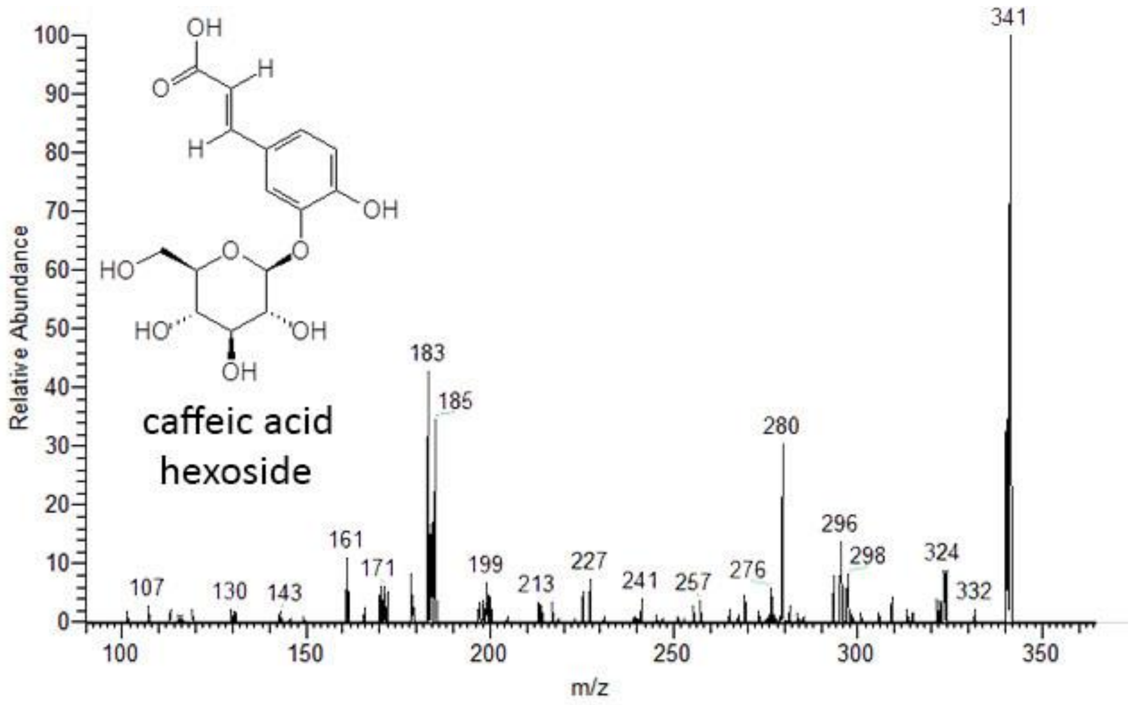

Figure S32. 5-O-caffeoylquinic acid PS(-)MS fragmentation and chemical structure.

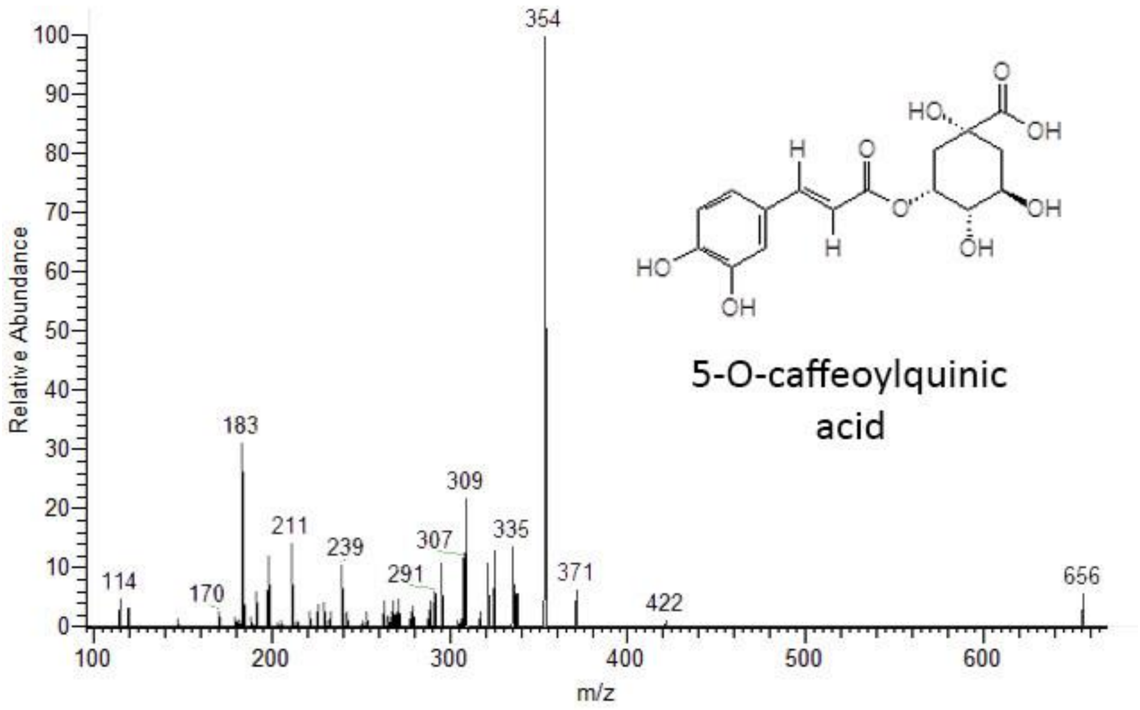

Figure S33. Kaempferol-O-pentoside PS(-)MS fragmentation and chemical structure.

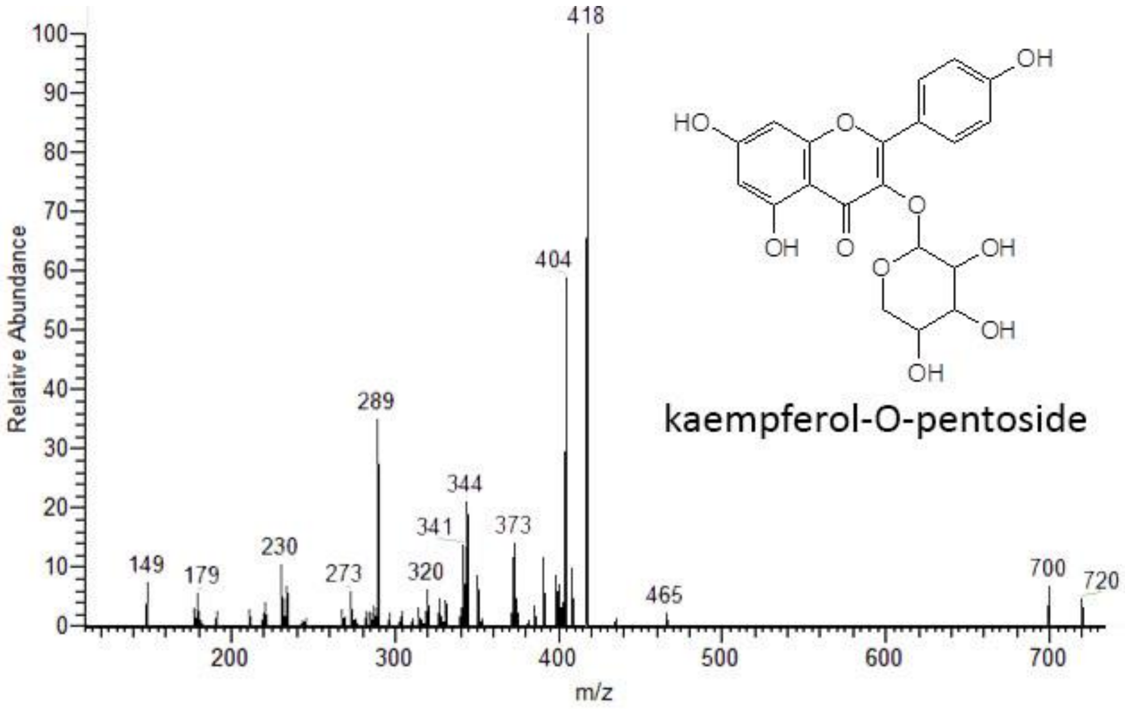

Figure S34. Cyanidin-3-O-arabinoside PS(-)MS fragmentation and chemical structure.

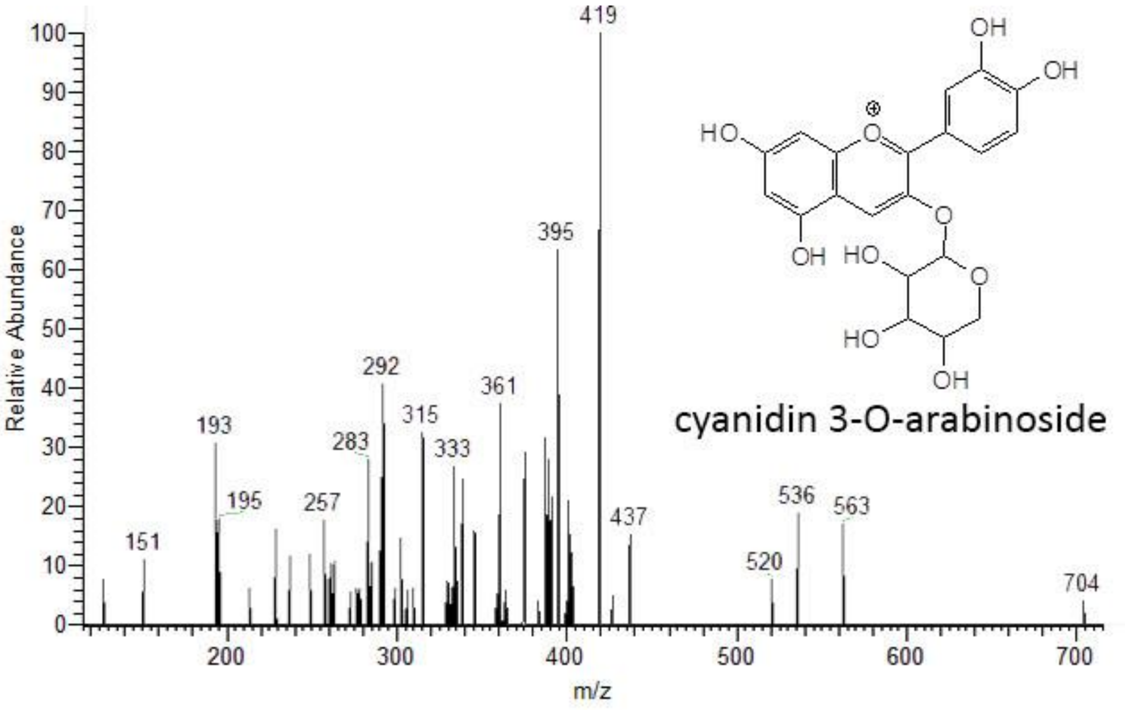

Figure S35. Vitexin PS(-)MS fragmentation and chemical structure.

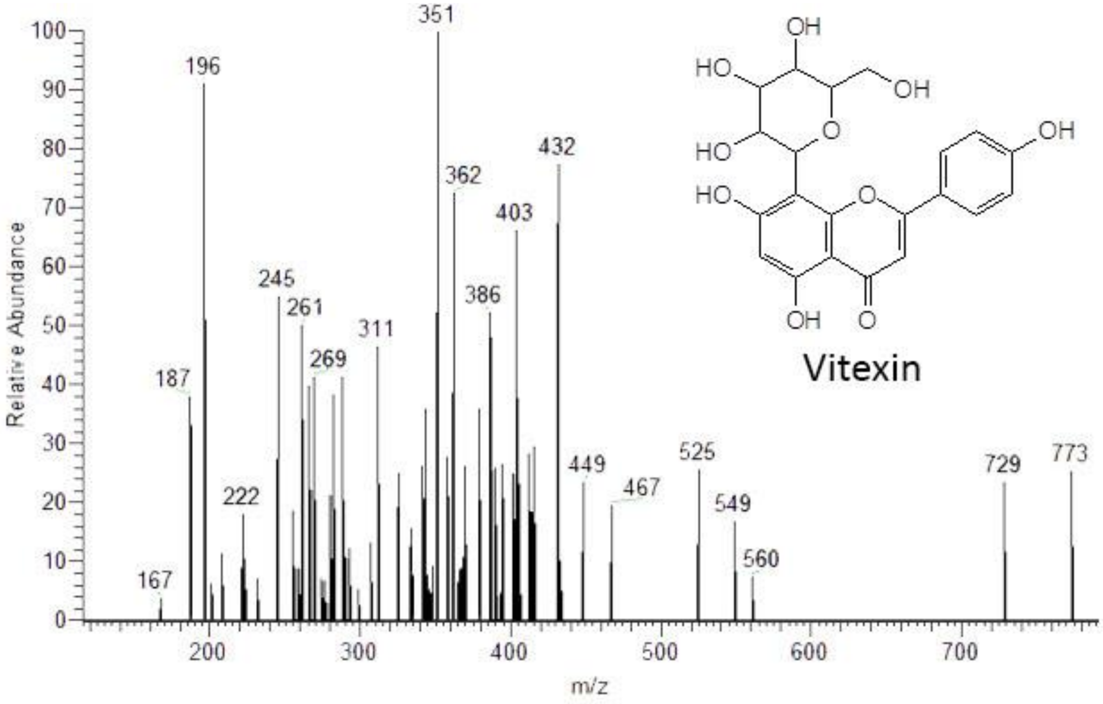

Figure S36. Peonidin-3-O-pentoside PS(-)MS fragmentation and chemical structure.

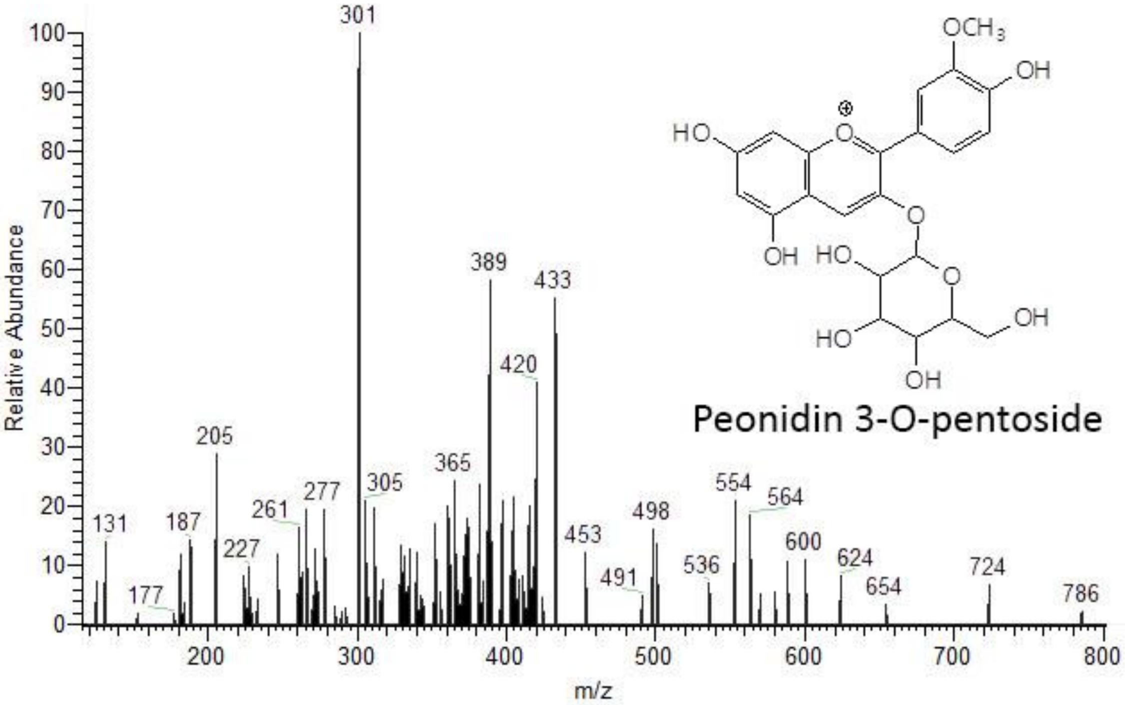

Figure S37. Phloridzin PS(-)MS fragmentation and chemical structure.

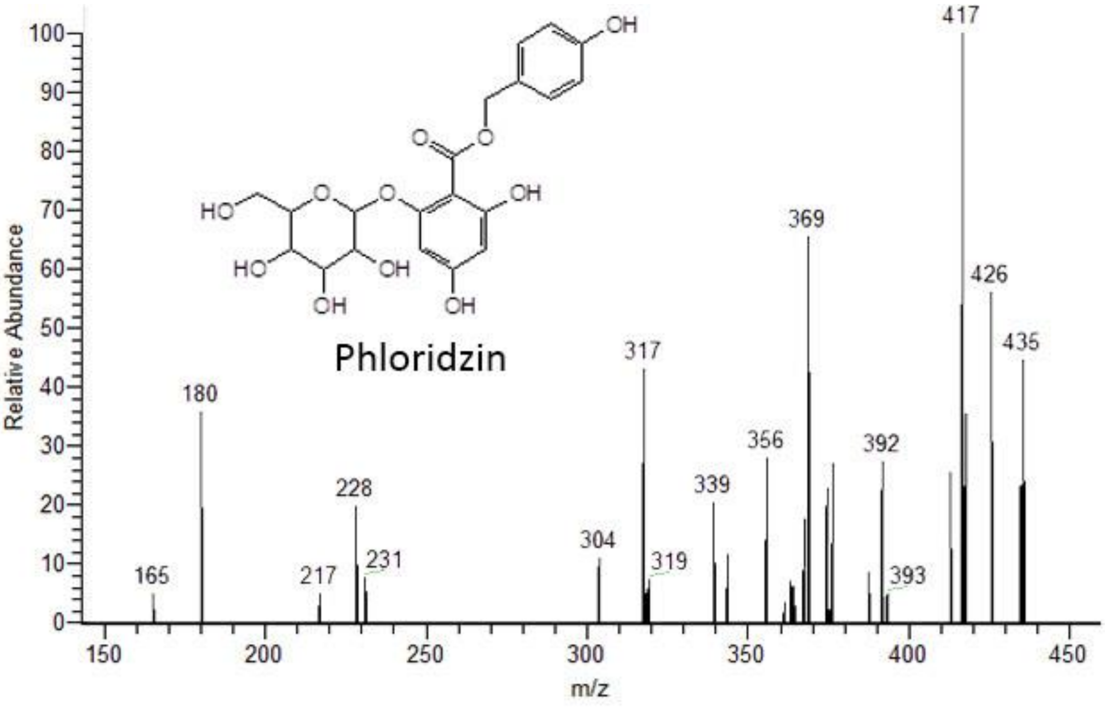

Figure S38. Kaempferol-O-hexoside PS(-)MS fragmentation and chemical structure.

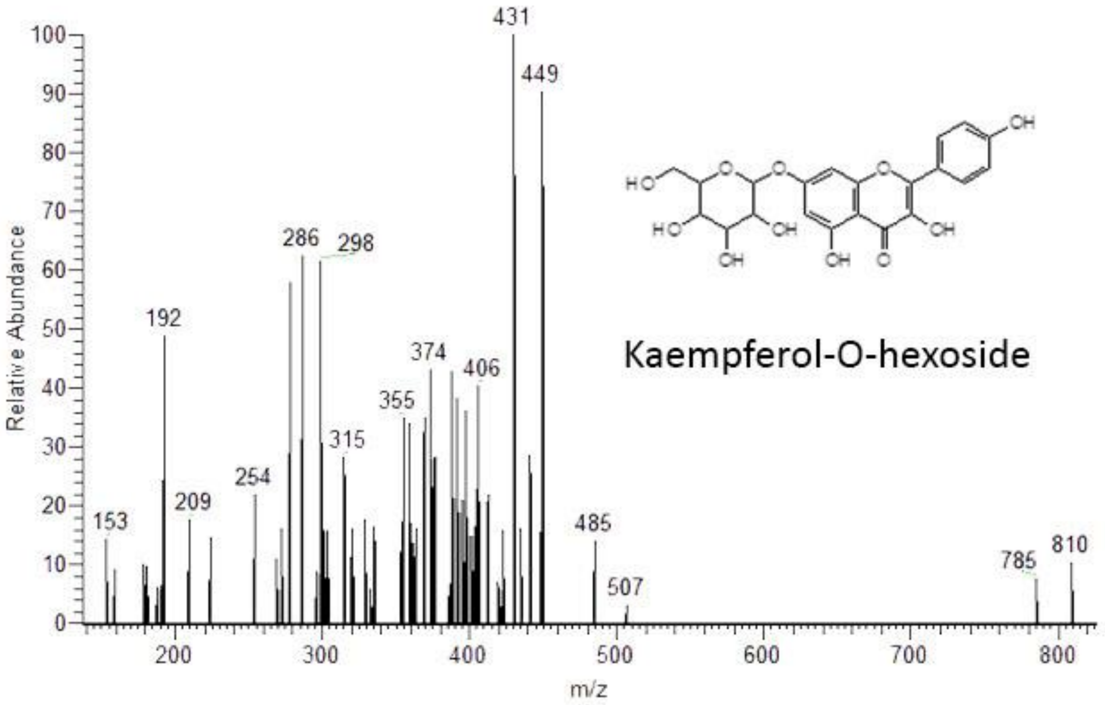

Figure S39. Dihydroquercetin-3,5-rhamnoside PS(-)MS fragmentation and chemical structure.

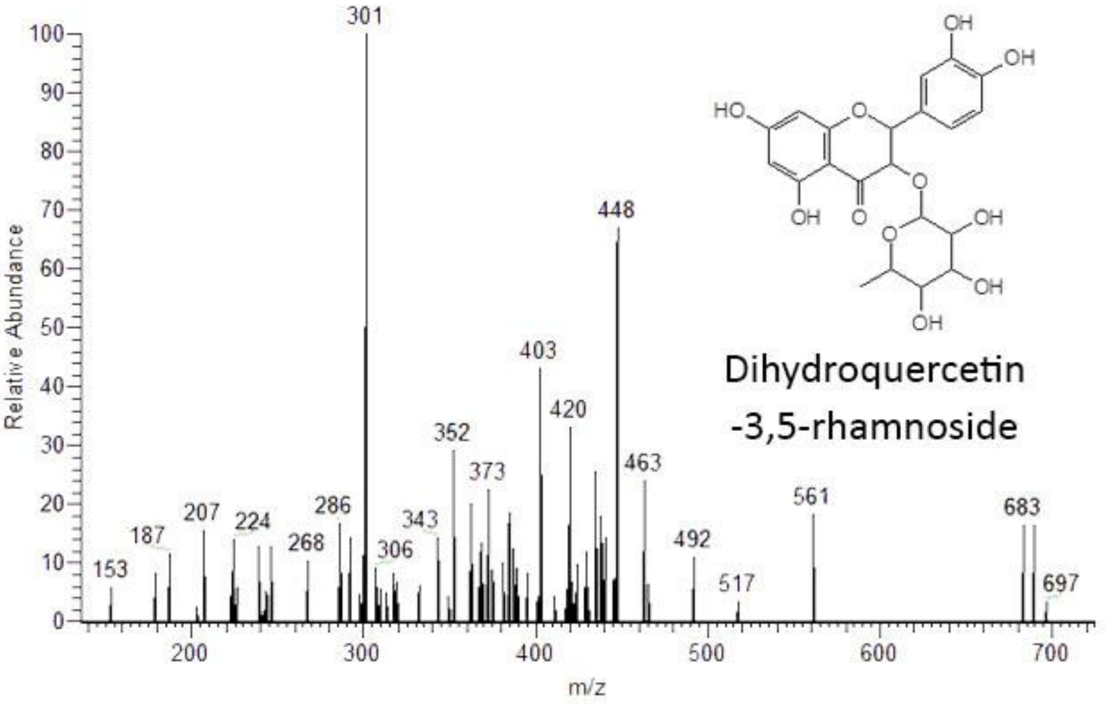

Figure S40. Isorhamnetin-O-coumaroyl PS(-)MS fragmentation and chemical structure.

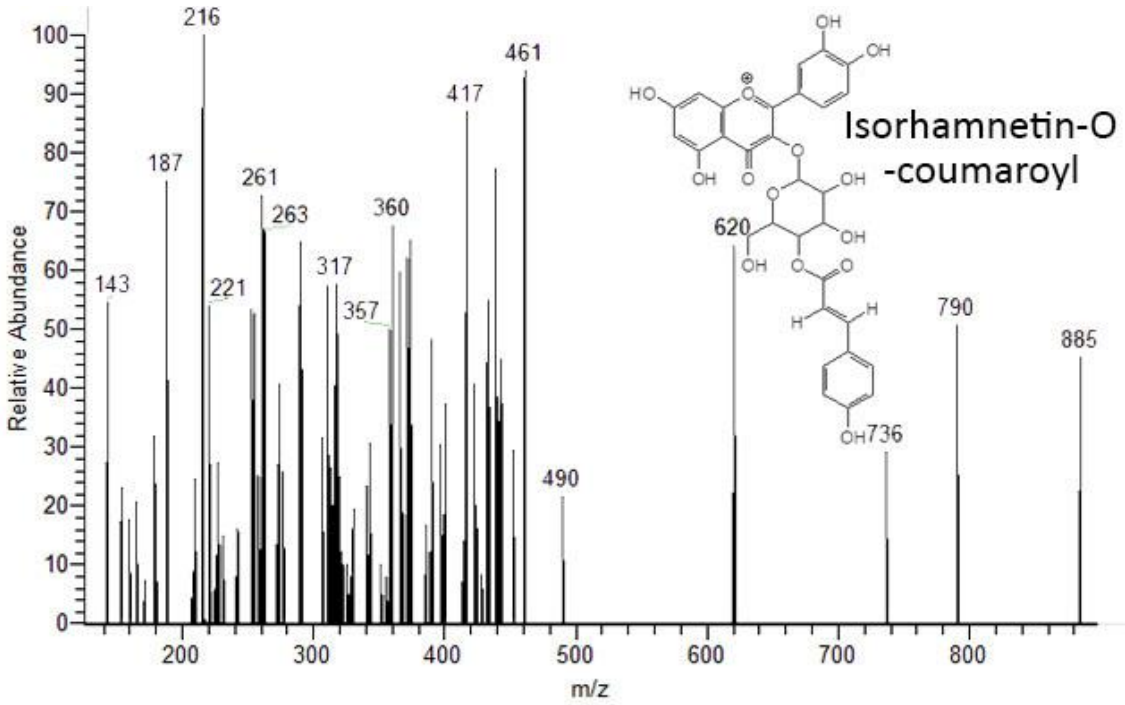

Figure S41. Quercetin-3-glucoside PS(-)MS fragmentation and chemical structure.

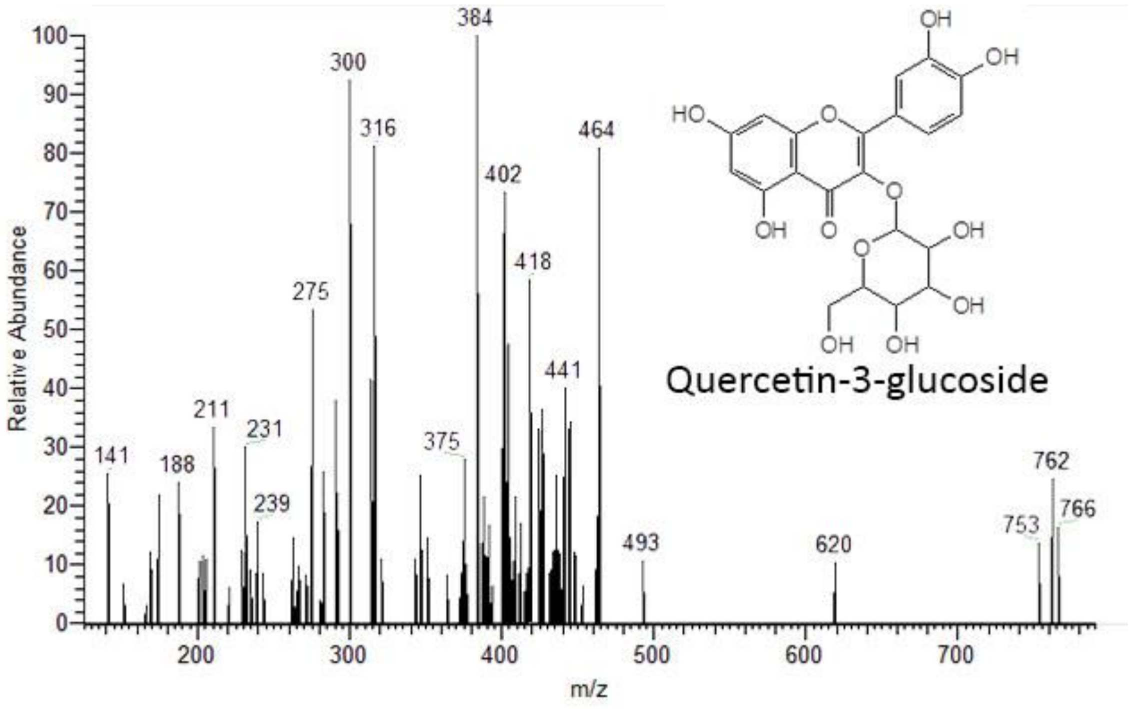

Figure S42. Quercetin glucuronide PS(-)MS fragmentation and chemical structure.

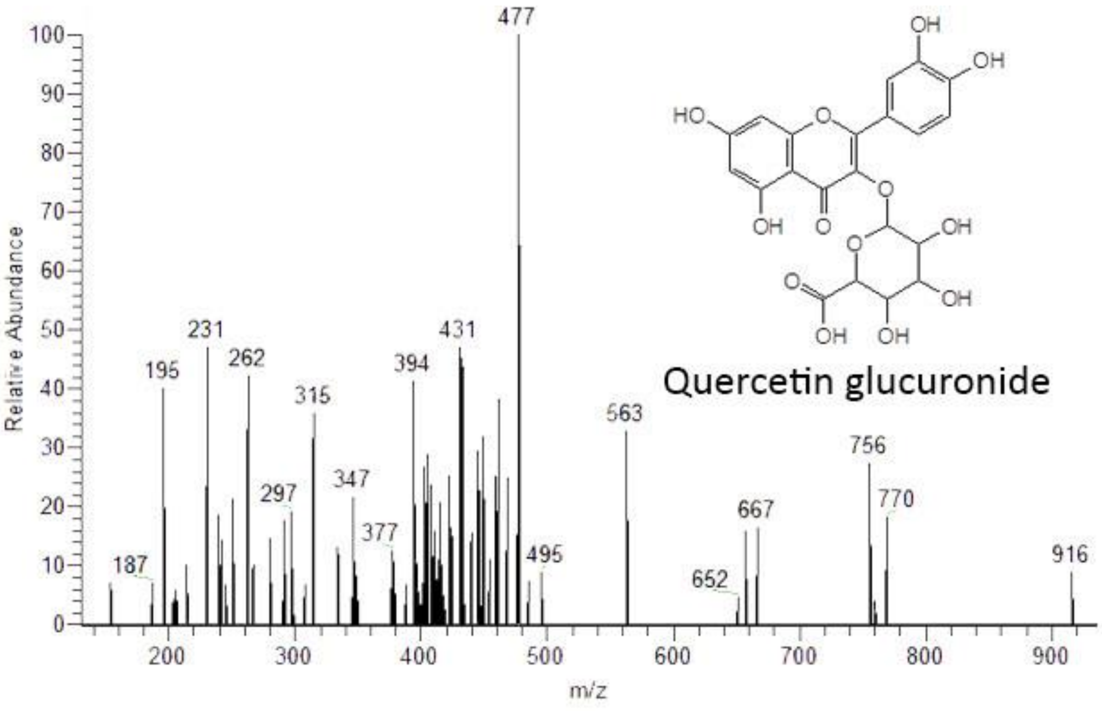

Figure S43. Caffeoyl hexose-deohyhexoside PS(-)MS fragmentation and chemical structure.

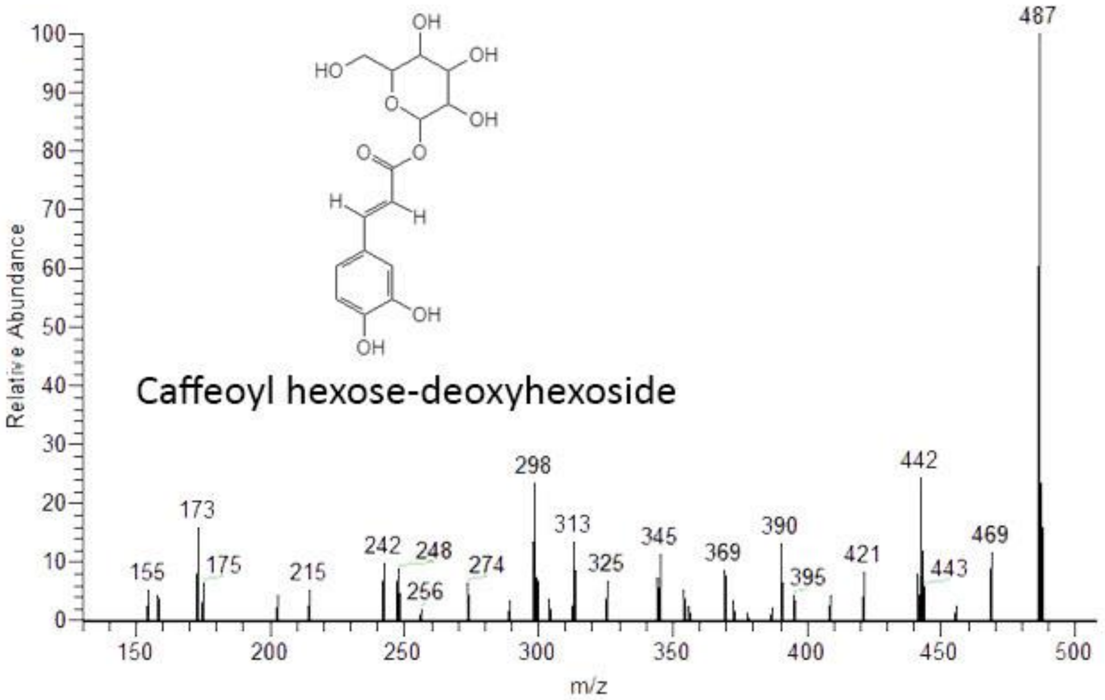

Figure S44. Dimethyl ellagic acid hexoside PS(-)MS fragmentation and chemical structure.

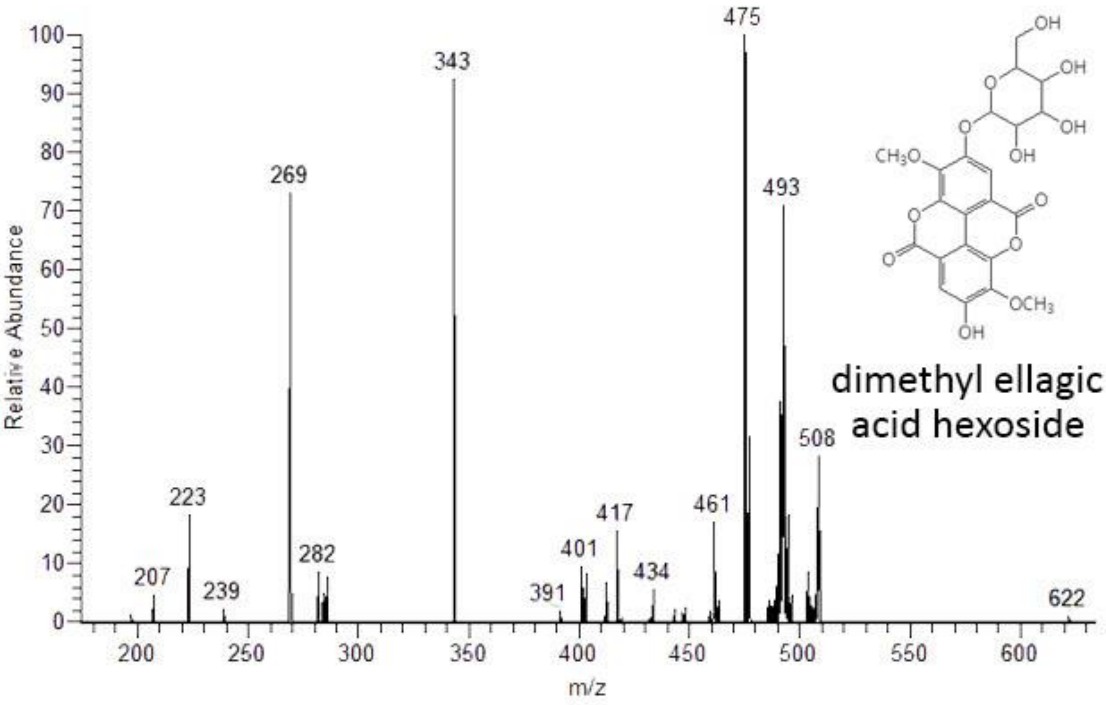

Figure S45. Apigenin-C-hexoside-C-pentoside PS(-)MS fragmentation and chemical structure.

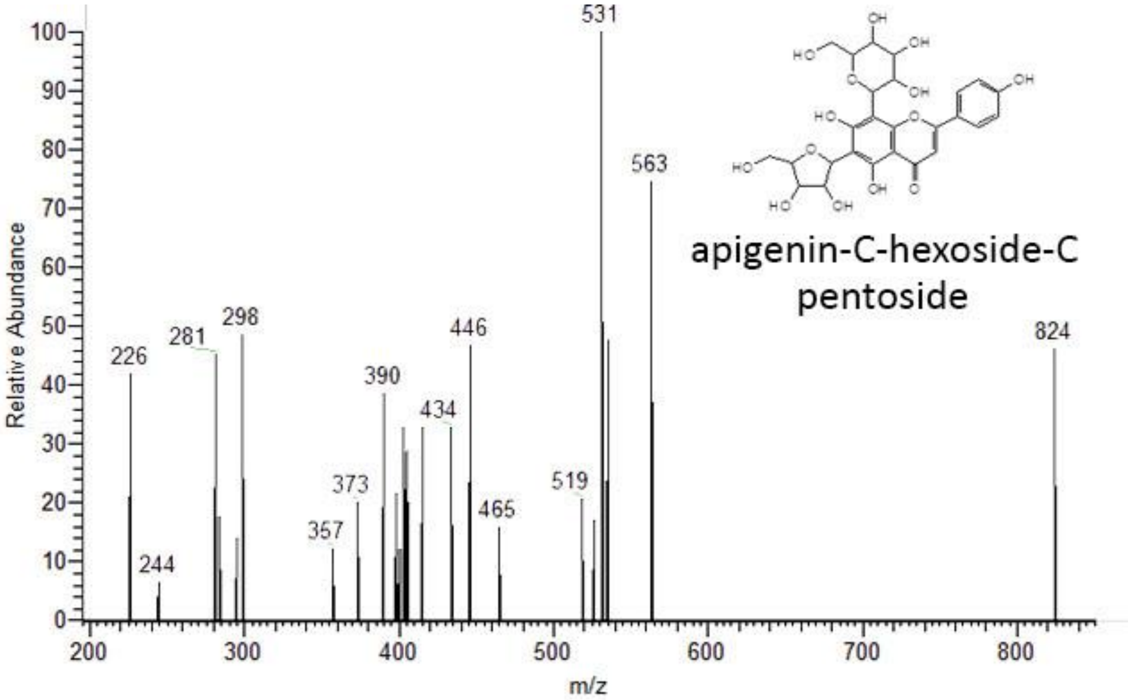

Figure S46. Luteolin-7-O-(2″-O-pentosyl)-hexoside PS(-)MS fragmentation and chemical structure.

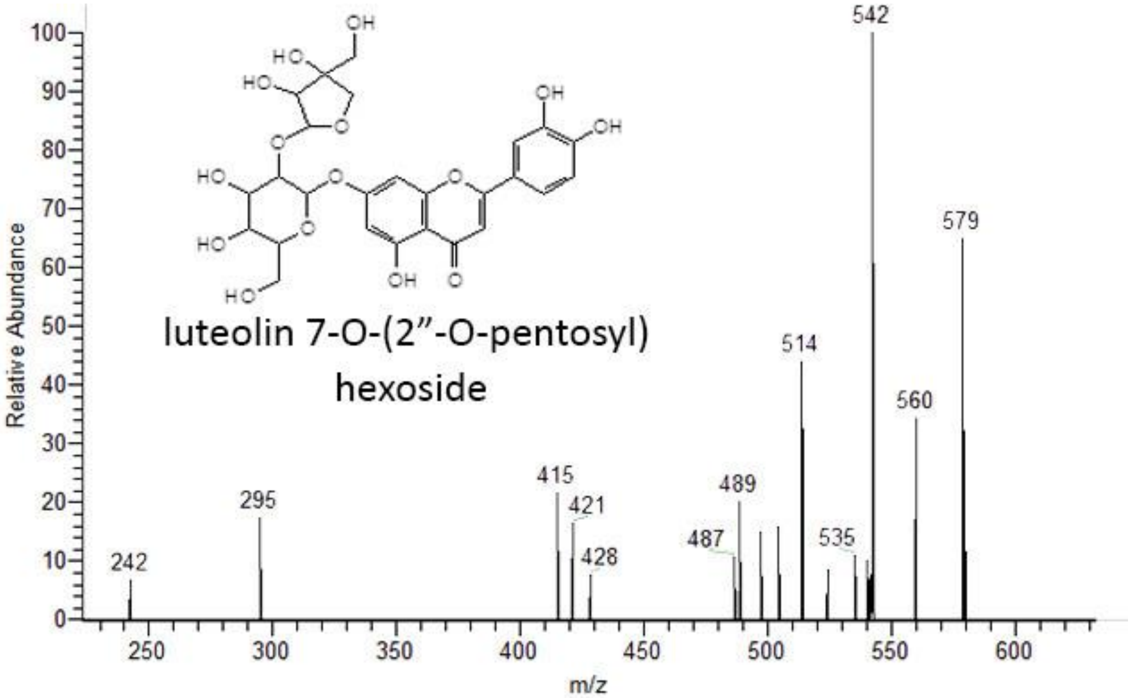

Figure S47. Catechin diglucopyranoside PS(-)MS fragmentation and chemical structure.

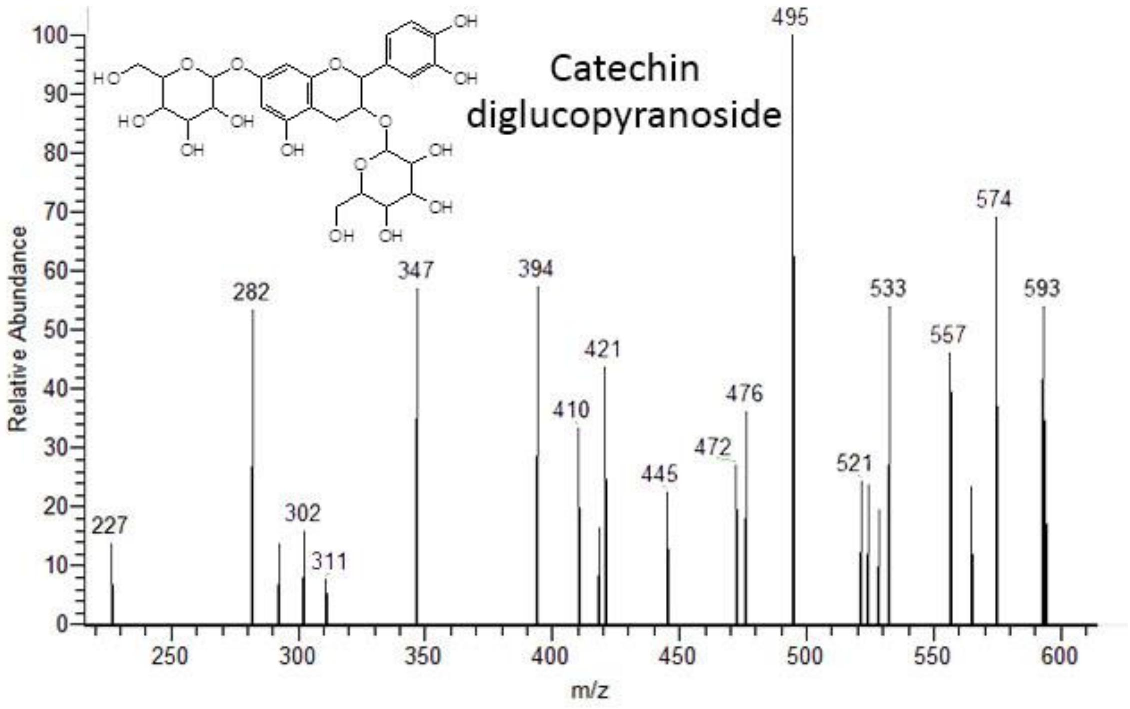

Figure S48. Quercetin-3,4′-O-diglucoside PS(-)MS fragmentation and chemical structure.

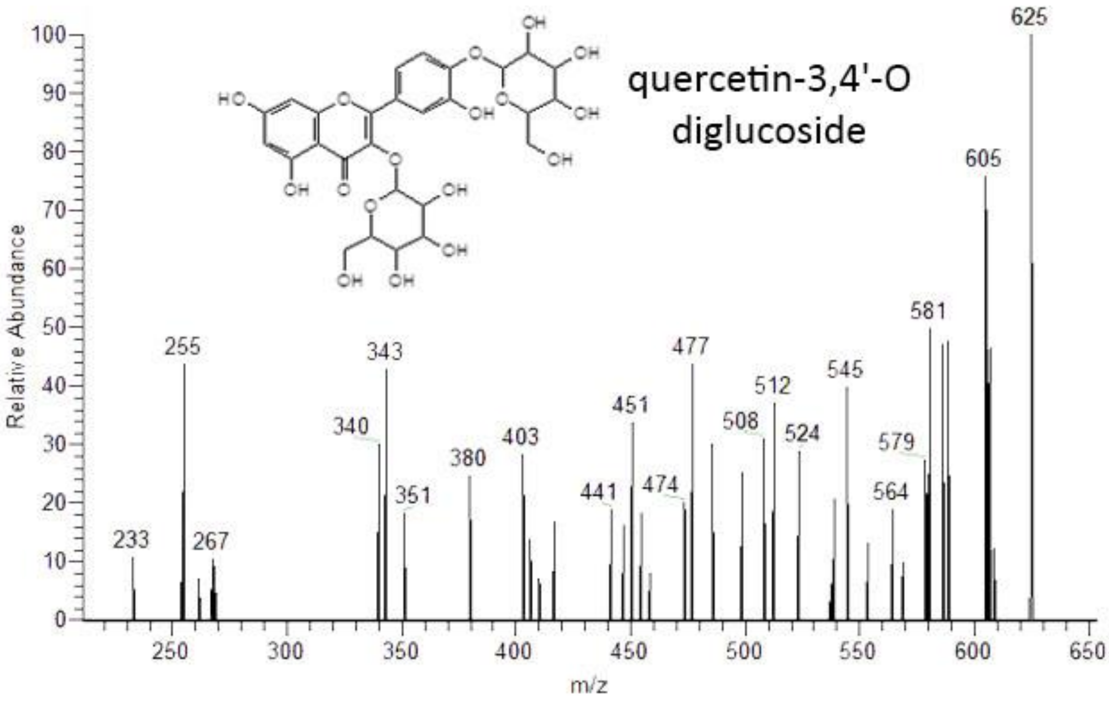

Supplement: Supplementary file 1 [file molecules-27-01892-s001.zip › molecules-1596606-supplementary.pdf]
